# Defining the extracellular matrix for targeted immunotherapy in adult and pediatric brain cancer

**DOI:** 10.1038/s41698-025-00956-z

**Published:** 2025-06-14

**Authors:** Zoe I. Day, Samuel Roberts-Thomson, Yasmin J. Nouri, Nathan S. Dalton, Stacie S. Wang, Alexander Davenport, Louise E. Ludlow, Mark D. Hulett, Ryan S. Cross, Misty R. Jenkins

**Affiliations:** 1 The Walter and Eliza Hall Institute of Medical Research, Parkville, VIC Australia; 2 Department of Biochemistry and Chemistry, School of Agriculture, Biomedicine and Environment, La Trobe Institute for Molecular Science, La Trobe University, Bundoora, VIC Australia; 3 Department of Anatomical Pathology, Royal Melbourne Hospital, Melbourne, VIC Australia; 4 The Department of Medical Biology, The University of Melbourne, Parkville, VIC Australia; 5 Children’s Cancer Centre, Royal Children’s Hospital, Melbourne, VIC Australia; 6 Murdoch Children’s Research Institute, The Royal Children’s Hospital, Flemington Road, Parkville, VIC Australia

**Keywords:** Tumour immunology, Cancer microenvironment, CNS cancer, Cancer, Drug discovery, Immunology

## Abstract

High-grade gliomas (HGGs), including glioblastoma (GBM) and pediatric diffuse midline gliomas (DMGs), remain highly fatal despite therapeutic advances. The tumor microenvironment (TME), particularly the extracellular matrix (ECM), plays a crucial role in tumor progression, immune exclusion, and drug resistance. We performed a comprehensive proteomic, transcriptomic, and pathological characterization of the ECM in primary adult and pediatric HGGs. Using cell surface proteomics, TCGA transcriptomics, and immunohistochemistry, we identified key ECM components influencing immune infiltration. We integrated these findings into *ImmunoTar*, a computational model prioritizing immunotherapeutic targets. Our study presents the first in-depth cell surface proteomic landscape of HGG ECM, identifying CSPG4/5, PTPRZ1, SDC1, TGFBR3, PLG, and GPC2 as key targets. We validate ECM-targeted CAR T cell therapy, including Glypican-2 (GPC2), which shows strong efficacy against pediatric DIPG. These findings highlight ECM-focused immunotherapy as a promising strategy to overcome HGGs’ immunosuppressive TME, particularly in pediatric patients.

## Introduction

High-Grade Gliomas (HGG) are a group of devastating brain tumors that include subtypes such as Glioblastoma (GBM), which comprises over 60% of all central nervous system (CNS) cancer cases in adults^1^. Despite decades of research, treatment options remain limited, and the prognosis for HGG patients continues to be poor, with a ~ 5% 5-year survival rate^1,2^. Similarly, pediatric High-Grade Gliomas (pHGG), including GBM (pGBM) and Diffuse Midline Gliomas (DMG), are regarded as universally fatal, making CNS tumors the leading cause of cancer-related deaths in children aged 0–14^1,3^. These dismal survival statistics highlight the urgent need for novel therapeutic strategies. The tumor microenvironment (TME) has been recognized as a formidable barrier to effective cancer treatment, playing a pivotal role in shaping immune responses and influencing therapeutic outcomes.

Cancer cells exploit normal homeostatic mechanisms to not only sustain their own growth and survival but also evade immune surveillance, creating a hostile and protected environment that hinders immune cell infiltration and activity. The bidirectional crosstalk between tumor cells and the surrounding stroma is a critical driver of cancer progression, significantly influencing disease trajectory and treatment response. Tumors actively recruit stromal, immune, and endothelial cells, leveraging secreted cytokines and chemokines to remodel the TME into an interconnected, immunosuppressive network.

One underexplored yet critical component of HGG biology is the extracellular matrix (ECM), a complex and dynamic structure composed of non-cellular macromolecules that provide structural and biochemical support to cells within tissues. Beyond its architectural function, the ECM plays an active role in tumor biology^4,5^, regulating cellular invasion^5^, immune exclusion^6^, drug resistance, and tumor progression^4,7^. The ECM consists of four primary macromolecule categories: proteoglycans^8–10^, glycoproteins^10–12^, fibrous proteins^10–12^ and glycosaminoglycans (GAGs)^8–10^, which together create a highly heterogeneous microenvironment across different tissues and disease states including cancer^10,11^. Unlike many cellular markers, the ECM is more stably expressed and is essential for tumor survival, making it a compelling yet largely underexplored target for therapeutic intervention.

The ECM constitutes approximately 20–30% of total brain volume and exhibits distinct characteristics compared to other tissues^13^. Unlike the ECM in other organs, the brain ECM contains lower levels of fibrous proteins (e.g., collagens and fibronectins) and is instead enriched in proteoglycans and GAGs^14^. Within the brain parenchyma, proteoglycans such as chondroitin sulfate proteoglycans (CSPGs) and heparan sulfate proteoglycans (HSPGs) are dominant and play key roles in neuronal development, cellular signalling, and tumor progression^9,10,13,14^.

Emerging evidence suggests that ECM remodeling plays an active role in shaping the tumor microenvironment (TME), influencing both tumor progression and therapeutic response. Recent transcriptomic studies have linked elevated collagen gene expression to GBM progression^15^, and GBMs have been shown to actively restructure the ECM to sustain their invasive and immune-excluded phenotype^16^. While certain ECM compositions have been implicated in tumor dormancy, a collagen-rich and stiffened ECM niche can also impede therapeutic penetration and efficacy^17^.

Chimeric antigen receptor (CAR) T cell therapy has emerged as a promising immunotherapy, offering targeted cytotoxicity against tumors by engineering T cells to recognize and eliminate malignant cells in an antigen-specific manner. CARs are synthetic fusion proteins that incorporate an antigen-binding domain derived from monoclonal antibodies, coupled with intracellular signaling domains that enhance T cell activation, proliferation, and persistence. While CAR T cell therapies have demonstrated remarkable success in hematologic malignancies, their efficacy in solid tumors, including HGGs, remains limited due to challenges such as antigen heterogeneity, physical barriers imposed by the tumor microenvironment, and immunomodulatory mechanisms.

Given the fundamental role of the ECM in HGG biology, we sought to comprehensively analyze the ECM composition in both adult and pediatric HGGs, recognizing its potential as an emerging therapeutic target. Here, we introduce the concept of the ECM as a novel library of tumor-associated antigens for CAR T cell therapy. Unlike cellular targets that can undergo antigen loss or downregulation, the ECM provides a tumor-specific, structurally integral, and functionally relevant set of antigens that can be exploited for immunotherapy. Additionally, the ECM exhibits significant interpatient variability, offering the potential for bespoke and personalized therapeutic approaches tailored to individual tumor profiles. Moreover, targeting ECM components has the potential to modulate the tumor microenvironment, disrupting immune exclusion mechanisms and enhancing the infiltration of other immune effector cells. In recent years, several groups have developed CARs targeting the ECM as a therapeutic approach to overcome the challenges posed by antigen heterogeneity and immune exclusion in solid tumors. These efforts have focused on ECM components such as collagens, hyaluronan and proteoglycans, which are critical for tumor progression and immune evasion. ECM-targeted CARs offer a dual advantage—they not only disrupt the structural support of tumors but also enhance T cell infiltration by modifying the tumor microenvironment. Preclinical studies have demonstrated promising efficacy of CAR T cells targeting ECM components, including Glypican-1 (GPC1)^18^, cerebroglycan or Glypican-2 (GPC2)^19–22^, Tenascin-C^23^, and Chondroitin Sulfate Proteoglycan 4 (CSPG4)^24^.

To explore this therapeutic potential, we conducted a comprehensive proteomic, transcriptomic, and pathological analysis of the ECM matrisome in primary adult and pediatric HGGs. Through immunohistochemical analyses, we examined ECM composition and deposition, assessing its variability within and between tumors. We employed cell-surface proteomics to identify tumor-specific proteoglycans and fibrous proteins. We analyzed our datasets with a recently developed computational tool, ImmunoTar^25^, designed to systematically rank and prioritize immunotherapeutic targets for cancer therapy. ImmunoTar enriched our cell surface proteomics datasets with quantitative parameters derived from multiple publicly available databases, carefully selected based on predefined criteria^25^. We further correlated our findings with transcriptomic data from The Cancer Genome Atlas (TCGA) to contextualize ECM signatures within a larger patient cohort. Whilst we acknowledge that there has been a generally poor correlation between mRNA and protein^26^, where correlations do exist, they can be leveraged in the development of novel therapeutics. To demonstrate this, we functionally validated the potential of ECM-targeting CAR T cell therapy by evaluating the efficacy of GPC2-targeting CARs against an adult and pediatric glioma model.

These findings provide the first in-depth cell surface proteomic characterization of the ECM in primary adult and pediatric HGGs, unveiling new potential therapeutic avenues for targeting the tumor microenvironment. By considering the ECM as a novel class of immunotherapy targets, this study lays the foundation for innovative treatment strategies aimed at overcoming tumor heterogeneity, enhancing immune infiltration, and improving therapeutic outcomes for these devastating malignancies.

## Results

The extracellular matrix (ECM) is a highly complex and dynamic network of macromolecules that provides both structural support and biochemical signals essential for cell function. In tumors, the ECM is continuously remodelled, promoting cancer progression, invasion, and immune evasion. However, due to the heterogeneity and redundancy of ECM proteins, traditional biomarker-driven approaches are insufficient to fully define targetable ECM components. To systematically address this gap, we applied an unbiased proteomic approach to identify novel ECM-associated targets that could serve as future candidates for immunotherapies.

### Proteomic characterization of ECM in pediatric DIPG identifies novel targets for immunotherapy

**Fig. 1:**
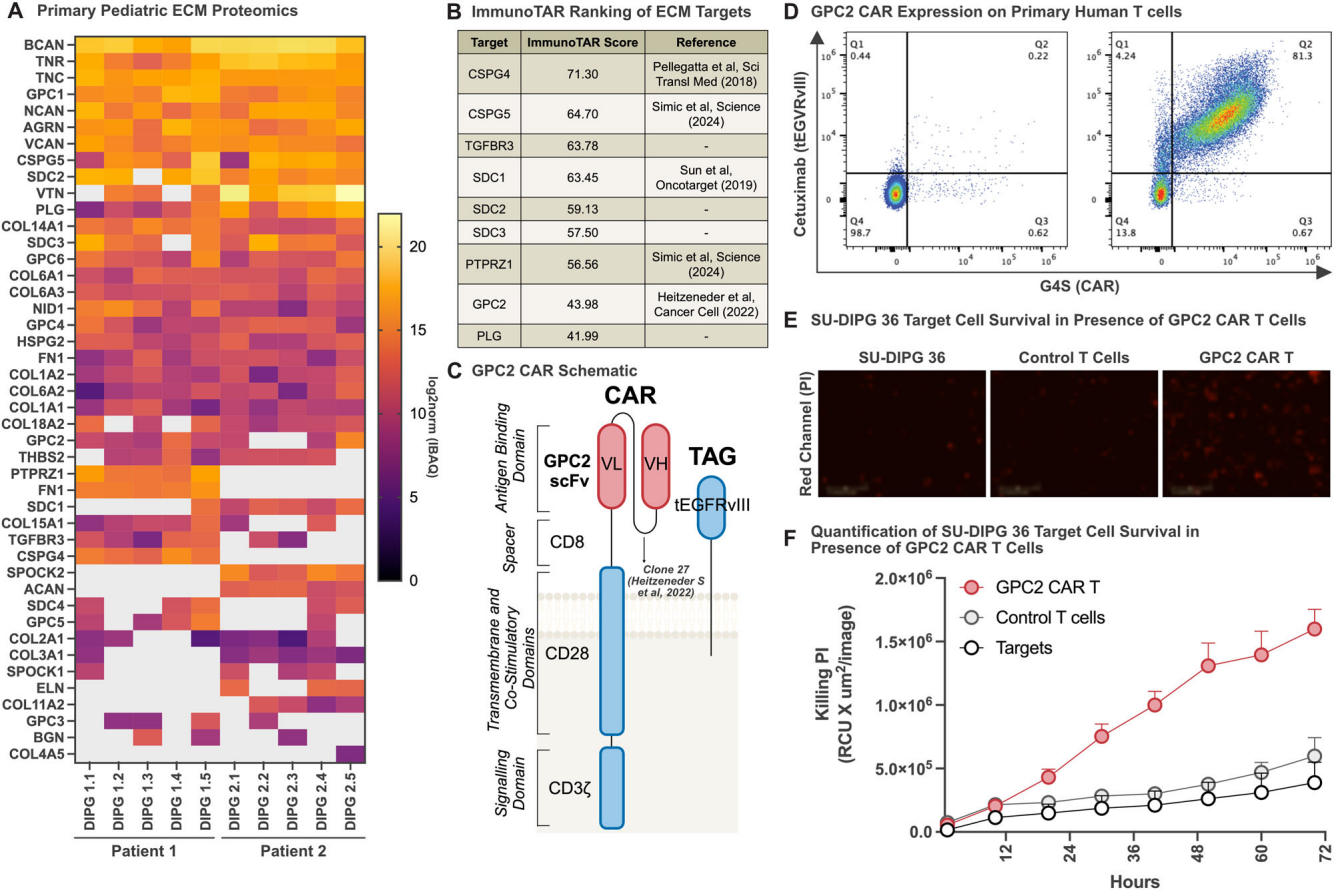
GPC2 CAR T cells are functional against pediatric high-grade glioma. **A** Cell surface proteomics of 2 primary pediatric diffuse intrinsic pontine gliomas (DIPGs) [5 replicates for each tumor] showing the presence of Extracellular matrix components detected at the cell surface (gray indicates lack of detection). **B** Top 9 Extracellular Matrix components based on pediatric DIPG cell surface proteomics for potential use as immunotherapy targets as predicted by an ImmunoTar score, with higher scores indicating stronger immune targeting potential. **C** Schematic of second generation GPC2 targeted CAR construct with truncated, non-signaling EGFRvIII cell surface tag. **D** Flow cytometry of cell surface expression of GPC2 CAR, as detected using anti-G4S protein tag antibody labeling (CAR) and cetuximab antibody (a-EGFRvIII), representative of two independent human donors. IncuCyte killing assay of 2:1 ratio of CD8 + GPC2-CAR T cells and control T cells co-cultured with pediatric SU-DIPG36GL tumor cells over 72 h, endpoint images and PI uptake shown in (**E**) (scale bar = 400 mm); quantification PI uptake over the 72 h in all conditions is shown in (**F**), respectively.

To define the ECM composition in pediatric Diffuse Intrinsic Pontine Glioma (DIPG), we analyzed two rare, primary DIPG tumors collected via rapid autopsy (*n* = 10 samples, five replicates per tumor, DIPG1 and DIPG2; Supplementary Table 1). We employed cell surface proteomics and mass spectrometry to generate an ECM-specific protein profile, identifying a comprehensive list of surface-exposed ECM components (Supplementary Tables 2 and 3), and present the most abundantly identified ECM proteins (Fig. 1A).

Through unbiased proteomic analysis we identified a diverse set of ECM-associated proteins that are highly expressed in pediatric high-grade gliomas (HGGs), with several proteoglycans, glycoproteins, and collagens emerging as potential immunotherapeutic targets. When interpreting the cell surface proteomic heatmaps, abundance comparisons should be made within individual columns rather than across rows, as each tumor sample’s IBAQ score and intensity are normalized within the respective column. Therefore, the data have been organized within columns to identify the most abundant ECM components in each sample throughout. Among these, Chondroitin Sulfate Proteoglycan 4 (CSPG4) was one of the highest-ranked proteoglycans, reinforcing its role as a tumor-associated antigen with prior validation as a CAR T cell target in glioblastoma^24^. Similarly, brevican (BCAN), an extracellular matrix glycoprotein highly expressed in gliomas, was identified as a top hit. Brevican is known to contribute to tumor invasion and ECM remodeling and has been demonstrated to be an ideal target for localized synthetic circuit induction^27^. Another notable ECM component, CSPG5, shares structural similarities with CSPG4 and is implicated neural development^28^, though it remains an underexplored target in CAR T cell therapy. CSPG5 has also been implicated as a prognostic marker in ovarian serous adenocarcinoma^29^. Additionally, Protein Tyrosine Phosphatase Receptor Type Z1 (PTPRZ1) emerged as a highly expressed ECM-associated target in pediatric HGG, which plays a key role in cell signaling, glioma progression, and immune modulation. PTPRZ1 has been shown to be overexpressed in glioblastoma stem cells and astrocyte-like tumour cells^30^ and while its expression is limited across adult tissues, it has been shown to be important in gliomagenesis^31,32^. Given its established function in tumor-stromal interactions, PTPRZ1 represents a compelling target for future immunotherapeutic strategies, and in fact some groups are also employing vaccine-induced approaches in GBM^30^. We identified Neurocan (NCAN) as one of the top 10 most abundantly expressed proteins (Fig. 1A), which has also been validated as a biomarker for glioblastoma by other groups^33^, and found to be expressed at low levels in healthy tissue^34^. NCAN has also been shown to provide a growth advantage to neuroblastoma^35^.

To further explore our proteomic dataset for cell surface targets, we applied ImmunoTar, a novel computational framework designed to systematically rank potential immunotherapeutic targets^25^. The ImmunoTar algorithm integrates our cell surface proteomic data with public datasets (e.g., GTEx, UniProt, DepMap) to generate a feature matrix characterizing each gene by tumor specificity, surface localization, and immunotherapy relevance. A weighted machine learning scoring algorithm ranks genes based on their suitability as immunotherapy targets, producing a prioritized list of candidate cell surface proteins for experimental validation. ImmunoTar analysis identified CSPG4, CSPG5, TGFBR3, SDC1, SDC2, SDC3, PTPRZ1, GPC2, and PLG as the highest-ranking extracellular matrix-associated proteins with strong potential as immunotherapy targets in pediatric high-grade gliomas (Fig. 1B).

Collectively, these findings provide a comprehensive roadmap for ECM-targeted immunotherapy, highlighting multiple promising targets that could be leveraged for CAR T cell therapy and combination approaches aimed at overcoming immune exclusion in gliomas.

### GPC2 as an oncofetal ECM-associated target in pediatric HGG

Next, we wanted to validate a candidate identified in the ImmunoTar analysis. Among the ECM components identified, Glypican-2 (GPC2) ranked eighth overall, reinforcing its potential as an immunotherapeutic target in pediatric HGG. GPC2 is a Glycosylphosphatidylinositol (GPI)-anchored proteoglycan that plays a key role in neural development, functioning as a signaling co-receptor regulating cell proliferation and differentiation^36,37^. While highly expressed during embryonic development, GPC2 expression is significantly downregulated in normal postnatal tissues. However, it has also been shown to be aberrantly overexpressed in pediatric neuroblastoma and other solid tumors^38,39^, making it a compelling target for CAR T cell therapy.

Previous studies have demonstrated the efficacy of GPC2-targeted CAR T cells in neuroblastoma and medulloblastoma^20,21^. Crystal Mackall’s group developed a suite of low-affinity GPC2-CARs, optimized for targeting tumors and decreasing toxicity, demonstrating efficacy in preclinical neuroblastoma models^19^. Expanding upon this work, Foster et al. validated mRNA transient GPC2-CAR T cell therapy in a xenograft model of medulloblastoma^22^. Therefore, while GPC2-directed CAR T cells have been previously explored, their effectiveness in targeting ECM-associated proteins within DIPG has not been previously described. Given our comprehensive ECM analysis in pediatric HGG, we sought to extend prior findings and first evaluate the utility of low-affinity GPC2-CAR T cells as part of a broader strategy to target ECM-associated proteins in DIPG.

### Validating efficacy of GPC2-directed CAR T cells in DIPG models

To assess the therapeutic potential of GPC2-targeted CAR T cells in DIPG, and to validate the target discovery pipeline, we selected the lowest-affinity GPC2 CAR construct to minimize on-target/off-tumor toxicity while maintaining tumor specificity^19^. Unlike previously validating the two higher-affinity GPC2 CARs^19^, the clone we chose had failed in targeting lower antigen density targets and we wanted to determine if it could achieve effective tumor targeting in DIPG given the high level of GPC2 detected in our proteomics.

Therefore, we engineered a second-generation GPC2-CAR with a CD28ζ intracellular signaling domain, and also included a truncated EGFRvIII tag for tracking transduced cells (Fig. 1C). Transduction efficiency was confirmed by direct labeling with an anti-G4S antibody which binds a small tag in the CAR and a cetuximab antibody that binds the EGFRvIII truncation tag, which showed robust CAR expression in CD8^+^ T cells (Fig. 1D). To evaluate cytotoxicity, patient-derived SU-DIPG36 tumor cells were co-cultured with GPC2-CAR T cells in an in vitro cytotoxicity assay (Fig. 1E). As anticipated, human donor-derived GPC2-directed CAR T cells demonstrated significant antigen-specific tumor cell killing relative to negative control donor matched untransduced T cells or target cells alone (Fig. 1F). While GPC2-CAR T cells have already demonstrated efficacy in neuroblastoma models, our findings support their inclusion as a potential therapy for pediatric HGGs.

### ECM characterization and immunotherapeutic targeting in adult glioblastoma

**Fig. 2:**
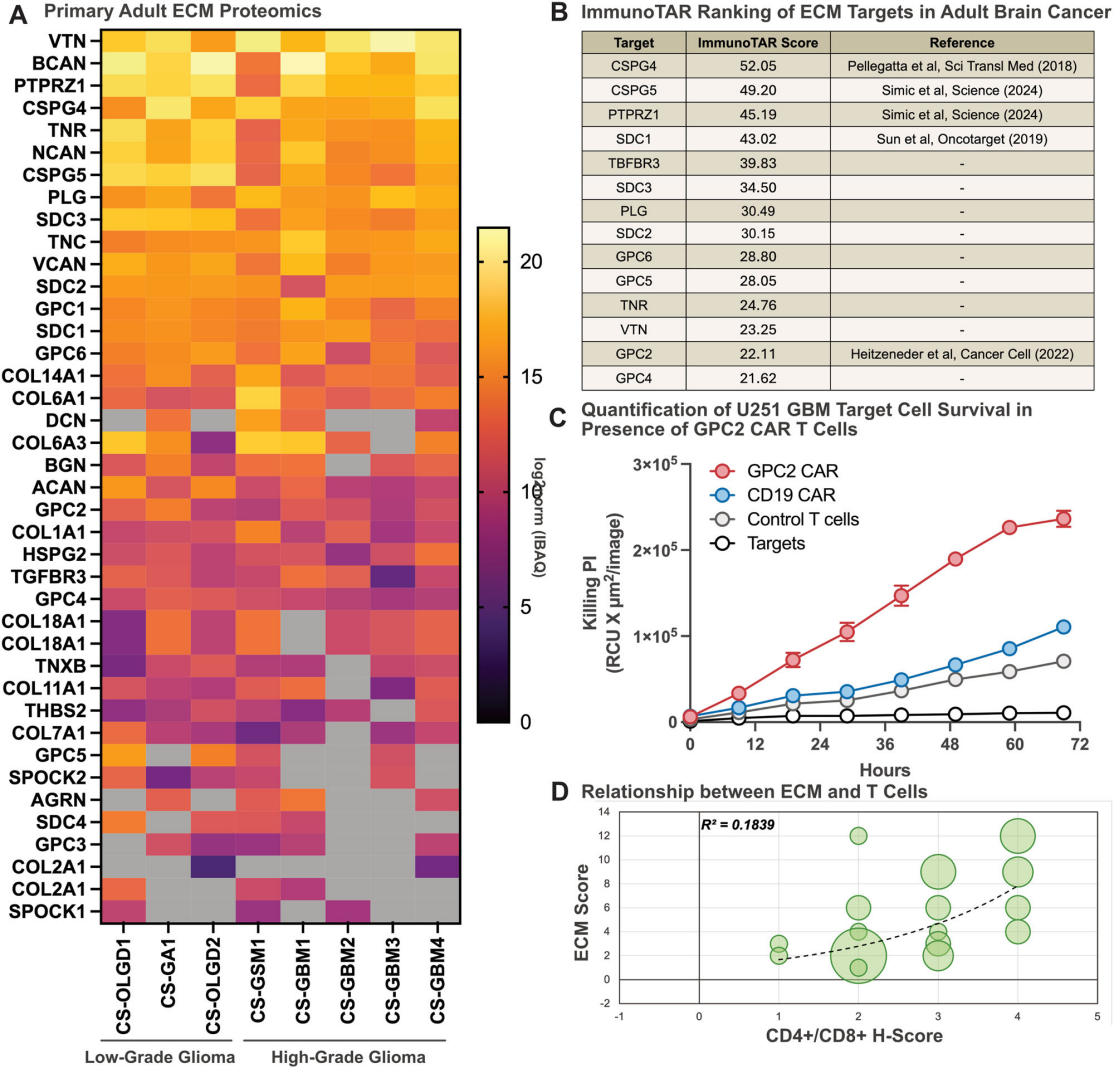
GPC2 CAR T cells are also functional against adult glioblastoma. **A** Cell surface proteomics of 8 primary brain cancers (including low and high-grade gliomas) showing the presence of Extracellular matrix components detected at the cell surface (gray indicates lack of detection). **B** Top 14 Extracellular Matrix components based on adult brain cancer cell surface proteomics for potential use as immunotherapy targets as predicted by an ImmunoTar score, with higher scores indicating stronger immune targeting potential. **C** Quantification of PI uptake over the 72 h of an IncuCyte killing assay of 2:1 ratio of previously described CD8 + GPC2-CAR T cells, CD19 negative control CAR T cells and control T cells co-cultured with pediatric glioblastoma U251 tumor cells over 72 h. Histology was performed on 40 primary Adult High Grade Glioma tumor samples [across a 30-patient cohort] to determine extend of endogenous T cells infiltration (dual anti-CD4+ and anti-CD8+ stains) and ECM deposition using Alcian Blue (stains glycosaminoglycans) and Massons Trichome (stains collagen) stains. Staining was performed on serial cut tumor sections and scored by an anatomical pathologist. **D** Here we show a bubble graph comparing T Cell infiltration (H-score from anti-CD4+ and anti-CD8+ stain) and ECM deposition (composite score of the Alcian blue H-score multiplied by the Massons Trichome H-Score) in adult brain cancers, the correlation between these two factors yielded a Pearson’s correlation coefficient of 0.184.

To extend our findings beyond pediatric HGGs, we next analyzed the cell surface proteome of primary adult brain cancer samples, including glioblastoma (GBM), to characterize their ECM composition (Fig. 2A). Using the same unbiased proteomic approach, we profiled 32 tumor samples from eight Grade II-IV glioma patients (four replicates per tumor; Supplementary Table 4). Filtering this dataset using established ECM-specific lists (Supplementary Tables 2 and 3) allowed us to generate a heatmap of the most abundant ECM components, identifying a number of similar proteins to that identified in pediatric HGG, such as VTN, BCAN, PTPRZ1, TNR, CSPG4 and NCAN as the predominant surface-expressed proteins (Fig. 2A). Vitronectin (VTN) emerged as the highest-ranking ECM component in the cell surface proteomic analysis of adult glioblastoma, highlighting its potential role in tumor progression, adhesion, and immune modulation. VTN has been shown to contributes to tumor cell survival, metastasis, and resistance to apoptosis across various cancers^40–43^. While VTN is overexpressed in GBM, it is also widely present in normal tissue and successful targeting strategies would need to differentiate tumour-associated VTN from circulating or stromal VTN, through methods such as genetically engineering T cell function to be restricted in the brain^27^.

We then applied the ImmunoTar analysis to the adult cell surface proteomics, which provided similar proteins predicted as good immunotherapy targets such as CSPG4, CSPG5, PTPRZ1 and GPC2 to those identified in the pediatric dataset (Fig. 2B). Given that GPC2 ranked as the 14th highest target in adult gliomas (Fig. 2B), we determined if low-affinity GPC2 CAR T cells could also target adult GBM, and therefore conducted functional testing against U251 GBM cells in an in vitro cytotoxicity assay. As anticipated, human donor-derived GPC2-directed CAR T cells demonstrated significant antigen-specific tumor cell killing relative to negative control donor-matched CD19 CAR T cells, untransduced T cells or target cells alone (Fig. 2C). These results support GPC2 as a viable immunotherapeutic target in adult glioblastoma. More broadly, our findings underscore the therapeutic potential and relevance of ECM components such as GPC2, CSPG4, and PTPRZ1 as novel pan-glioma immunotherapy targets.

To evaluate the relationship between ECM composition and endogenous T cell infiltration, we performed immunohistochemical labeling of CD4^+^ and CD8^+^ T cells (Supplementary Fig. 1) and stratified the results based on the ECM score. The ECM score was calculated as the sum of Alcian Blue (GAG staining) and Massons Trichome (collagen staining) H-Scores, reflecting the overall extent of ECM deposition by quantifying GAG and collagen presence, respectively.

Consistent with previous studies, we found that HGGs are predominantly immune-cold tumors with limited T cell infiltration^44–46^. However, upon analysis which returned a Pearson correlation coefficient of 0.184, a weak correlation was observed between ECM score and T cell presence (Fig. 2D), suggesting that a more structured ECM may support immune cell recruitment. One possible explanation is that specific ECM components retain chemokines and adhesion molecules, fostering a microenvironment conducive to immune cell accumulation^47^. However, a larger sample cohort would be needed to confirm these findings.

These findings underscore the dual role of the ECM in both structural support and immune modulation, emphasizing the need for further studies that could enhance T cell infiltration and improve immunotherapy efficacy in gliomas.

### Glycosaminoglycan deposition and ECM proteomics in adult and pediatric gliomas

**Fig. 3:**
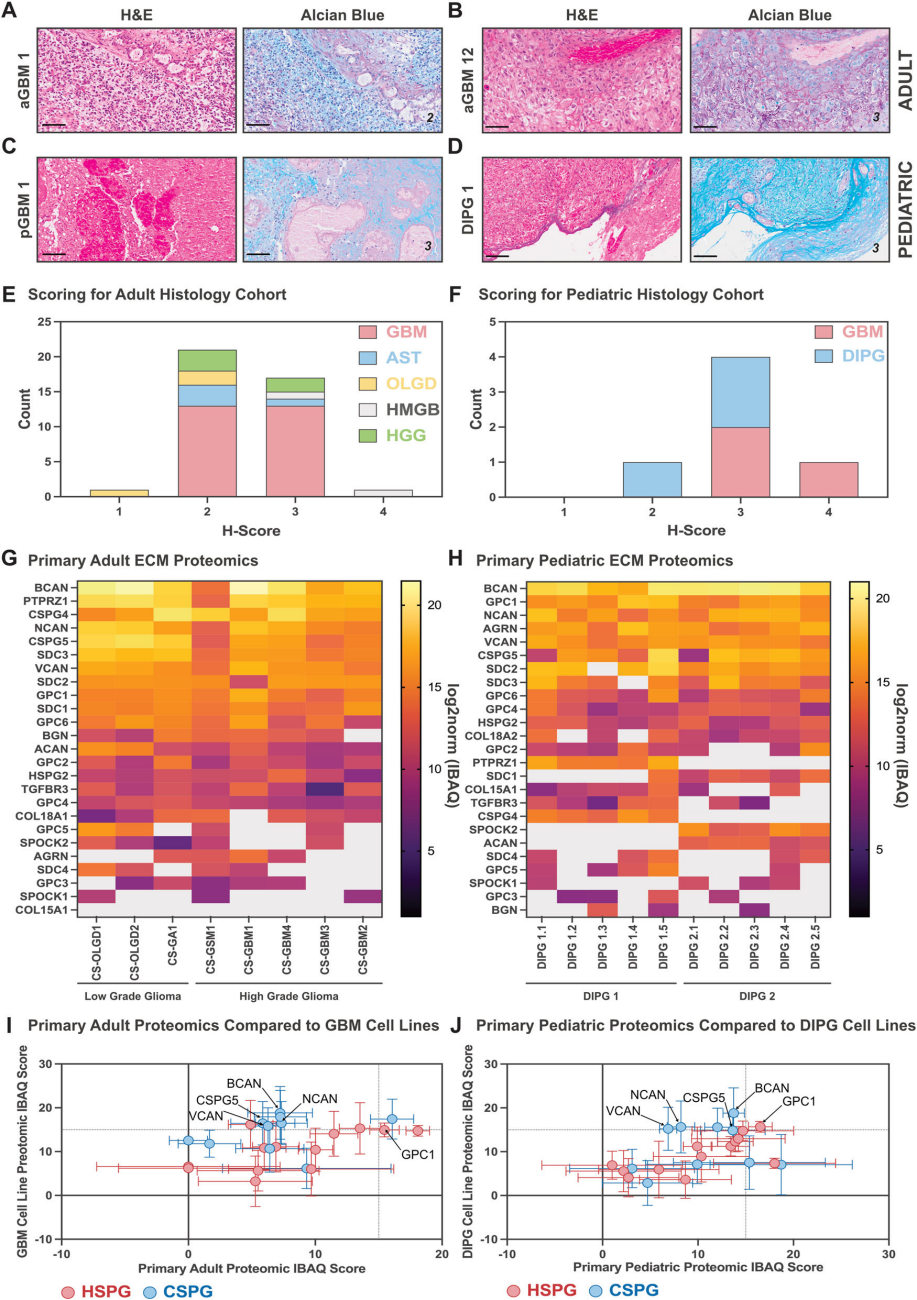
Adult and pediatric brain cancers contain high levels of targetable extracellular matrix proteoglycans. Histology was performed on 40 primary Adult High Grade Glioma tumor samples [across a 30-patient cohort] and 6 rare primary pediatric high-grade gliomas and scored by an anatomical pathologist. Staining was performed on serial cut tumor sections, here we show Alcian Blue (stains glycosaminoglycans) staining and the corresponding H&E sections from (**A**, **B**) two different adult glioblastomas (aGBM) (score 2 and score 3, respectively) and two pediatric high-grade gliomas including (**C**) a pediatric glioblastoma (pGBM) (score 3) and an (**D**) Diffuse Intrinsic Pontine Glioma (DIPG) (score 3) (scale bar = 100 μm); The scoring of alcian blue, and presence of glycosaminoglycans, staining for adult and pediatric cohorts, by diagnosis, is quantified in (**E**) and (**F**), respectively; Cell surface proteomics of (**G**) 8 primary adult brain cancers (including low and high-grade gliomas) and (**H**) 2 primary pediatric diffuse intrinsic pontine gliomas (DIPGs) [5 replicates for each tumor] showing the presence of targetable Heparan Sulfate Proteoglycans (HSPGs) and Chondroitin Sulfate Proteoglycans (CSPGs) detected at the cell surface (gray indicates lack of detection); Comparison of the presence (mean IBAQ scores) of HSPGs and CSPGs identified by cell surface proteomics in both primary human brain cancers and model cell lines used in preclinical models with specific comparisons between (**I**) primary adult brain cancers (low and high grade gliomas) and GBM cell lines (U87, SW1088, U118, H4, A1172 and U251) and (**J**) primary pediatric DIPG tumors and patient-derived DIPG model cell lines.

To further investigate the extent and distribution of ECM components, we performed Alican Blue (AB) staining to assess glycosaminoglycan deposition (Fig. 3A–D). Six pediatric HGG samples were included (Supplementary Table 1) and thirty adult brain tumor samples were analyzed, with some patients providing biological replicates, resulting in a total of 40 adult tumor sections (Supplementary Table 5). Staining intensity and distribution were evaluated and scored by an anatomical pathologist, generating a scoring system which ranked samples from high to low glycosaminoglycan deposition (Supplementary Fig. 2).

### Glycosaminoglycan deposition across adult and pediatric gliomas

AB staining revealed robust and consistent glycosaminoglycan deposition across all tumor samples, with all adult and pediatric sections showing positive staining (Fig. 3A–D, Supplementary Tables 6 and 7). Quantitative analysis showed that the majority of adult glioma sections (38/40) exhibited moderate glycosaminoglycan deposition, with scores of 2 or 3, across all tumor subtypes (Fig. 3E). A similar pattern of glycosaminoglycan deposition was observed in pediatric tumors (Fig. 3F). Notably, stratification based on tumor grade or patient sex did not reveal additional insights. Importantly, all analyzed sections primarily contained tumor tissue without identifiable tumor borders, reinforcing that moderate glycosaminoglycan deposition occurs within the tumor core in both adult and pediatric gliomas.

### Proteomic analysis of glycosaminoglycan-associated ECM components

Given the moderate to high levels of glycosaminoglycan deposition, we next sought to characterize the specific ECM composition using LC-MS/MS cell surface mass spectrometry proteomics. Since glycosaminoglycans predominantly bind to core proteins to form proteoglycans^8,9^, we analyzed mass spectrometry data to determine which proteoglycans were present in primary HGG samples. This included heparan sulfate proteoglycans (HSPGs), chondroitin sulfate proteoglycans (CSPGs), keratan sulfate proteoglycans (KSPGs), and dermatan sulfate proteoglycans (DSPGs) (Supplementary Table 2).

Across both adult (Fig. 3G) and pediatric gliomas (Fig. 3H), BCAN (brevican) was the most abundantly detected proteoglycan. Comparing the top 10 most abundant proteins, we identified NCAN, VCAN, CSPG5, and GPC1 in both adult and pediatric tumors, suggesting conserved ECM protein expression across age groups. The absence of peptide detection in mass spectrometry does not necessarily indicate a lack of expression, but may be due to variability in detection sensitivity and sample-specific abundance. However, our analysis focused on the most abundantly detected ECM components.

### Comparison of primary tumor ECM profiles with in vitro cell lines

Since 50% of the top 10 ECM proteins were shared between adult and pediatric gliomas, we next validated whether these proteins were similarly expressed in glioma cells in vitro. Cell surface proteomics was performed on adult and pediatric human glioma cells, and protein expression patterns were compared to those observed in respective primary tumor samples (Fig. 3I, J, Supplementary Fig. 3 and 4).

Interestingly, adult glioma cell lines displayed protein expression profiles that deviated from those observed in primary tumors, with several proteins detected in vitro but not in primary tumor tissues (Fig. 3I). In contrast, pediatric glioma cell lines exhibited greater concordance with primary tumors, suggesting a more faithful representation of in vivo ECM composition (Fig. 3J). This discrepancy in adult tumor samples could be due to greater tumor heterogeneity in primary glioblastoma compared to the more uniform nature of pediatric DMG tumors, which were the primary subtype analyzed in this study.

These findings highlight both similarities and differences in ECM composition between adult and pediatric gliomas. While moderate glycosaminoglycan deposition was consistently observed in both groups, proteomic analysis revealed a partial overlap in ECM protein expression between age groups, with pediatric tumors displaying greater alignment between in vitro and in vivo ECM profiles. These data emphasize the need for age-specific models of glioma ECM biology and suggest that pediatric glioma cell lines may provide more reliable preclinical models for ECM-targeting therapies compared to adult glioblastoma lines.

### Collagen deposition varies between adult and pediatric gliomas

**Fig. 4:**
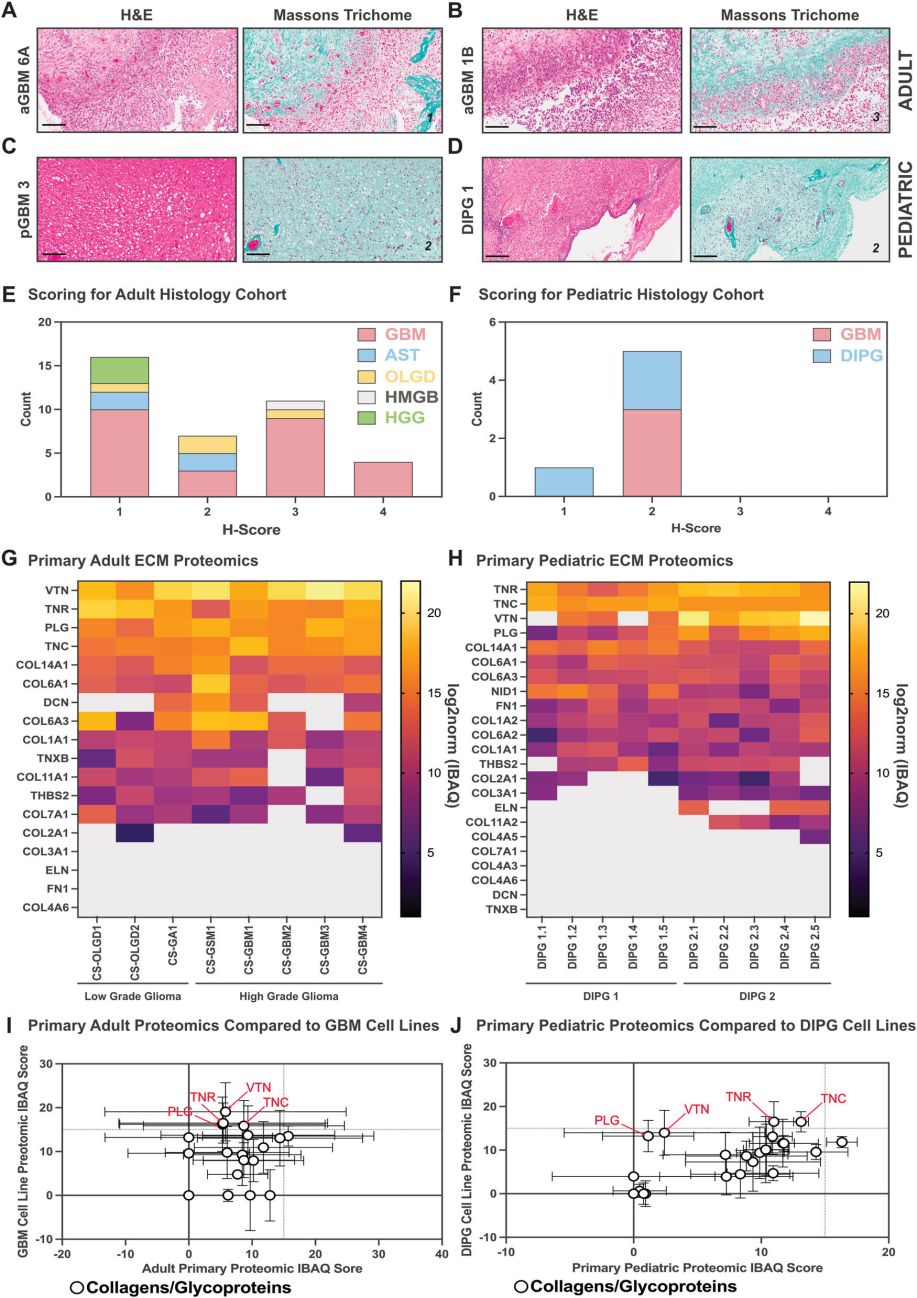
Collagen and other extracellular matrix glycoproteins are diffuse throughout adult and pediatric brain cancers. Like previously described histology was performed on primary adult and pediatric brain cancers, here we show Massons Trichome (stains collagen) staining and the corresponding H&E sections from (**A**, **B**) two different adult glioblastomas (aGBM) (score 1 and score 3, respectively) and two pediatric high-grade gliomas including (**C**) a pediatric glioblastoma (pGBM) (score 2) and an (**D**) Diffuse Intrinsic Pontine Glioma (DIPG) (score 2) (scale bar = 100 μm); The scoring of Massons Trichome, and presence of collagen, staining for adult and pediatric cohorts, by diagnosis, is quantified in (**E**) and (**F**), respectively; Cell surface proteomics of (**G**) 8 primary adult brain cancers (including low and high-grade gliomas) and (**H**) 2 primary pediatric diffuse intrinsic pontine gliomas (DIPGs) [5 replicates for each tumor] showing the presence of collagens and other extracellular matrix glycoproteins detected at the cell surface (gray indicates lack of detection); Comparison of the presence (mean IBAQ scores) of collagens and glycoproteins identified by cell surface proteomics in both primary human brain cancers and model cell lines used in preclinical models with specific comparisons between (**I**) primary adult brain cancers (low and high grade gliomas) and GBM cell lines (U87, SW1088, U118, H4, A1172 and U251) and (**J**) primary pediatric DIPG tumors and patient-derived DIPG model cell lines.

To further investigate the composition of core ECM proteins, we examined collagen deposition in both adult and pediatric gliomas using Masson’s trichrome (MT) staining (Fig. 4A–D). Historically, the brain has been considered to have low levels of fibrous ECM components, including collagen, and brain tumors were therefore believed to contain minimal collagen within their tumor microenvironment (TME) and ECM^10,11^. However, consistent with recent findings^15^, our analysis revealed collagen deposition in both adult and pediatric gliomas, as evidenced by positive MT staining (Fig. 4A–D).

To quantify collagen expression, an anatomical pathologist scored collagen deposition across tumor samples (Supplementary Fig. 5). In adult gliomas, collagen expression showed significant intertumoral variability, with heterogeneous distribution across tumor subtypes (Fig. 4E, Supplementary Table 6). By contrast, pediatric tumors exhibited consistently lower collagen staining (Fig. 4F, Supplementary Table 7). While our sample size is limited, this variation in collagen deposition suggests that targeting collagen-associated ECM components in gliomas may require tumor-subtype-specific therapeutic approaches.

### Identification of collagen subtypes and glycoproteins in primary gliomas

Having established collagen presence in both adult and pediatric HGGs, we next examined specific collagen isoforms and associated glycoproteins (Supplementary Table 3). Proteomic analysis of primary adult tumor samples identified fourteen ECM proteins, including seven distinct collagen isoforms (Fig. 4G). Among these, vitronectin (VTN) emerged as the most abundantly expressed ECM protein. VTN is known to play a role in neurogenesis in normal brain development^48,49^ and has also been implicated in GBM progression, tumor cell invasion, and integrin signaling pathways that contribute to cancer cell survival and resistance to apoptosis^50,51^ (Fig. 4G).

In the pediatric HGG (pHGG) cohort, eighteen collagens and glycoproteins were identified, with Tenascin R (TNR) emerging as the most abundant (Fig. 4H). TNR is highly expressed in the central and peripheral nervous systems^52^, consistent with its prevalence in pediatric gliomas. Interestingly, Tenascin C (TNC), another tenascin family member, was also highly abundant. While TNC expression is typically spatiotemporally restricted in normal tissues, it is known to be upregulated in GBM and other cancers, contributing to tumor progression and immune evasion^53–55^.

The collagen and glycoprotein profiles in pediatric HGGs showed partial overlap with adult tumors, with three of the top glycoproteins in adult gliomas (VTN, TNR, and PLG) also among the most highly expressed proteins in pediatric gliomas. Additionally, COL14A1 emerged as the most abundant collagen isoform in both pediatric and adult samples (Fig. 4G, H). These findings reinforce the complex ECM landscape of HGGs and suggest that specific ECM components could serve as immunotherapeutic targets to disrupt tumor-stroma interactions and tumor progression through shaping the tumour microenvironment.

### Proteomic profiling in GBM and patient-derived xenograft (PDX) cell models

To further explore these findings, we conducted the same proteomic analysis on adult glioma cells lines (Supplementary Fig. 6) and ten pediatric glioma cell lines (Supplementary Fig. 7). In adult, similar to the earlier analysis of proteoglycans, adult glioma cell lines displayed collagen and other glycoprotein expression that that differed from those observed in primary tumors (Fig. 4I, Supplementary Fig. 6). This analysis revealed a strikingly different collagen and glycoprotein profile, nearly reversing the abundance seen in primary tumors. In particular, COL6A1 was notably abundant, suggesting that it is possible that ECM components may be secreted into the ECM by stromal cells rather than tumour cells, which could account for the reduced abundance seen in 2D cell cultures compared to primary 3D tumor tissues. In contrast, pediatric glioma cell lines shared a higher abundance of collagens and other ECM glycoproteins with primary DIPG samples (Fig. 4J, Supplementary Fig. 7). ECM components like TNC and TNR shared similar expression profiles in both an in vitro and in vivo setting, highlighting again that while no model may be completely accurate in representing the true ECM of HGGs, pediatric cell-based models have the potential to be more biologically relevant than adults.

These findings highlight the cellular and molecular heterogeneity of the ECM in both adult and pediatric gliomas and strongly suggest that the composition of the ECM in glioma is derived from both tumour and stromal cells. While some ECM proteins are consistently expressed across tumor subtypes, others exhibit patient-specific intertumoral variability, reinforcing the need for targeted, tumor-subtype-specific therapeutic strategies. The differential ECM composition observed in in vitro models compared to primary tumors further emphasizes the importance of using physiologically relevant models when developing ECM-targeted therapies for gliomas.

### RNA and protein concordance in ECM-associated CAR T cell targets

**Fig. 5:**
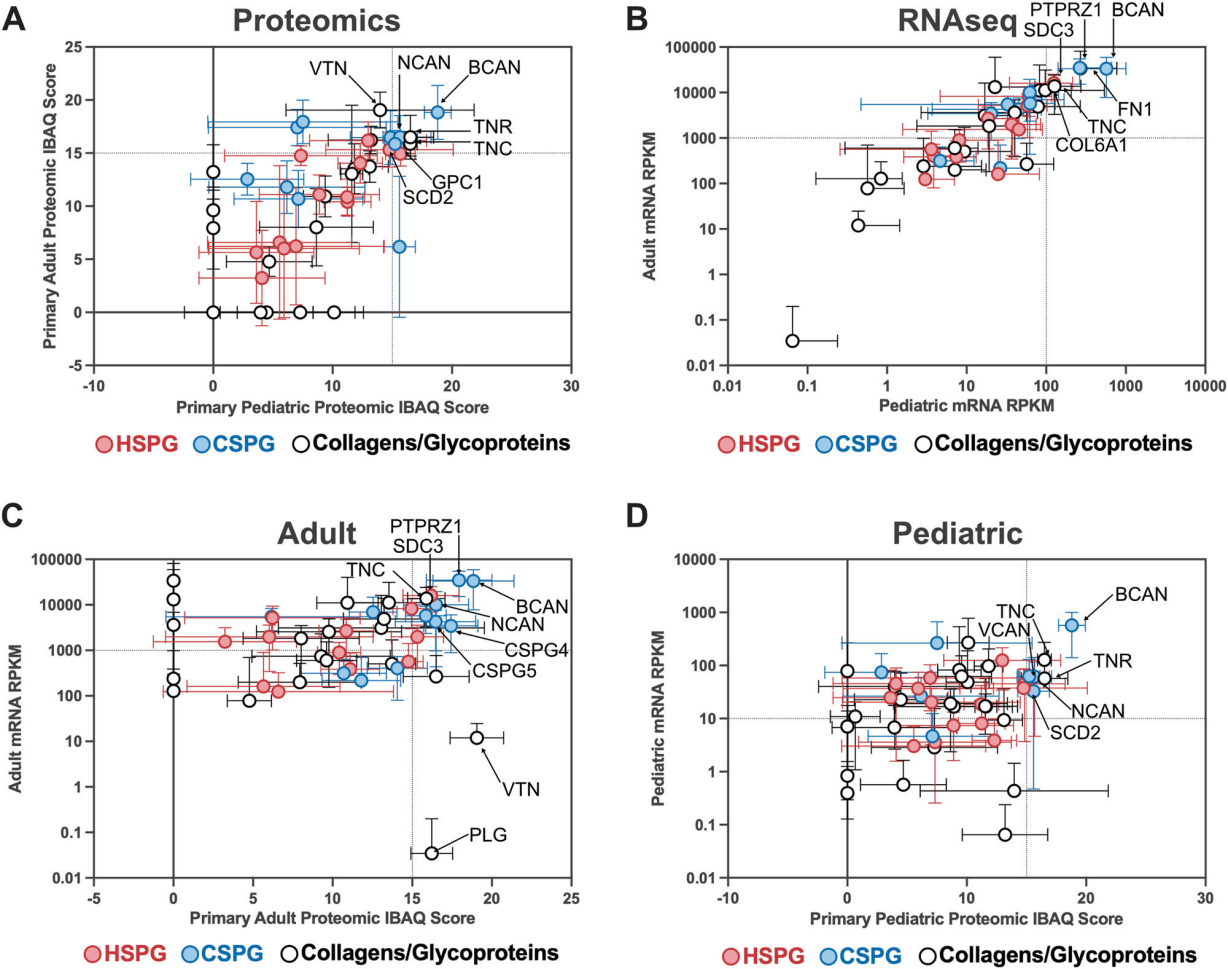
Correlation and identification of targetable extracellular matrix components. Analysis of cell surface proteomic data of primary adult and pediatric brain cancers and RNAseq data extracted from the TCGA data portal for both Adult Glioblastoma [Glioblastoma Multiforme (TCGA, PanCancer Atlas) *n* = 160 tumors] and Pediatric DIPG [Pediatric Brain Tumor Atlas (PBTA, Provisional) *n* = 1945 tumors] for correlation of protein and expression levels of extracellular matrix Heparan and Chondroitin Sulfate proteoglycans, collagens and glycoproteins. **A** Comparison of protein expression levels, based on cell surface proteomics previously shown, between primary adult and pediatric brain tumors. **B** Correlation of TCGA RNAseq data for ECM components between adult glioblastoma and pediatric DIPG. **C** Comparison of protein expression levels in primary adult brain tumors and the RNA expression levels from the TCGA glioblastoma data set. **D** Comparison of protein expression levels in primary pediatric DIPG brain and the RNA expression levels from the TCGA DIPG data set.

To further validate our findings and identify potential ECM targets shared between adult and pediatric gliomas, we compared the cell surface protein expression levels of ECM components detected in primary samples from adult and pediatric tumors (Fig. 5A). We found that the top concordantly expressed proteins were BCAN, TNC, TNR and GPC1. Given the limited sample size of our proteomic dataset and limitations of the sampling process whereby not all proteins can be successfully detected via mass spectrometry, we want to determine if correlations of expression could be found in larger RNA sequencing (RNAseq) expression data cohorts. Given the concordance we observed within our proteomic data, we next compared RNAseq expression data for the identified ECM components to determine how transcriptional data aligned between pediatric and adult gliomas (Fig. 5B) (for RNAseq data refer to Supplementary Data 1 [adult] and Supplementary Data 2 [pediatric]). This analysis is important to draw conclusions on targets that could be used across both age cohorts, as we know brain development greatly influences both tumor and brain composition. The pediatric brain is more plastic and rapidly developing, with a higher proportion of gray matter and ongoing synaptic pruning, whereas the adult brain has more established neural connections, greater myelination, and reduced plasticity^56–58^. However, the comparative analysis of adult and pediatric gliomas revealed an unexpected concordance in ECM gene expression, similar to our proteomics, suggesting that key ECM proteins may be consistently expressed at both the transcriptomic and proteomic levels. While it is well established that RNA and protein expression do not always correlate, identifying targets with high concordance between transcriptomic and proteomic datasets is particularly valuable for CAR T cell therapy development. Such targets ensure consistent antigen availability, which is critical for therapy efficacy and patient selection criteria in clinical trials.

To refine our candidate selection and determine if concordance between transcriptomic and proteomic data existed, we compared RNAseq expression with protein abundance in both adult (Fig. 5C) and pediatric glioma samples (Fig. 5D). This analysis identified CSPG4, PTPRZ1, and BCAN as the most highly expressed and concordant ECM targets in adult gliomas, while GPC1, CSPG5, and TNR were the top concordant targets in pediatric gliomas. Notably, existing CAR T cell therapies have already been developed against CSPG4^24^, BCAN^27^ and GPC1^18^, reinforcing their therapeutic relevance. In addition, TNR has been identified as a tumor-associated ECM protein with roles in cell adhesion, invasion, and immune evasion, and given its restricted expression in the central nervous system and involvement in glioma progression, it has been identified as a potential immunotherapy target^59^.

Given these findings, we propose that CSPG4, PTPRZ1, BCAN, GPC1, CSPG5, and TNR should be prioritized for further investigation as CAR T cell targets in glioma, particularly using combination approaches targeting multiple ECM components to enhance novel therapeutic strategies in gliomas.

## Discussion

Recent advancements in single-cell and spatial proteomics have further enhanced the ability to dissect ECM composition at an unprecedented resolution, uncovering molecular interactions critical for cancer progression and therapy resistance^60^. Integrating multi-omic datasets with advanced computational tools, such as machine-learning-driven target prioritization, provides a powerful framework for identifying high-value ECM therapeutic targets that can be exploited in emerging immunotherapies and engineered T cell therapies^61^. As the field evolves, unbiased proteomic approaches will be crucial for mapping tumor ECM landscapes and guiding spatial-omics approaches, ultimately accelerating the development of precision-targeted immunotherapies designed to overcome tumor microenvironment-mediated therapy resistance.

The comprehensive immunohistochemical and surfaceome analyses conducted in our studies provide valuable and detailed insights into the extracellular matrix (ECM) composition of both adult and pediatric high-grade gliomas (HGG) and its implications for tumor biology, including therapeutic target identification and development. Here, through the detection and analysis of different families of proteoglycans, collagen isoforms and other ECM-associated glycoproteins, across large cohorts of patients with primary HGG, we have been able to demonstrate that, like cells that make up these aggressive tumors, the ECM is dynamic and heterogenous, demonstrating patient-to-patient variability. We also acknowledge that out findings may also not be completely definitive, with more tumor-associated ECM components yet to be uncovered, due to limitations with proteomic processing as not all ECM proteins may be usefully detected during mass spectrometry due to technical factors like peptide flight efficiency. Our findings do however support the current paradigm shift that views the ECM as more than just structural support, and more as a dynamic component of the tumor microenvironment that influences tumor progression, invasiveness, and response to therapies^16^. This study also departs from previous understandings of HGG conceptualized the brain and brain tumors as being devoid of fibrous ECM components like collagen, which we have found to be present in both an adult and pediatric brain tumors. A recent study by Watson et al. (2024) reveals that multiple therapies targeting glioblastoma can induce a fibrotic response in the tumor microenvironment, which, paradoxically, promotes tumor cell survival and recurrence. Through multi-omics analysis, the authors identify fibrosis as a pro-survival niche that inhibits immune surveillance, suggesting that targeting fibrosis-associated pathways could improve the efficacy of anti-glioma therapies^62^.

Both heparan and chondroitin sulphate proteoglycans, including notable components like Syndecan-1 (SDC1)^63–65^, Glypican-1 (GPC1)^66–68^ and Brevican (BCAN)^69–71^ found in this study, have previously been identified to be integral in mediating tumor invasiveness and poor patient outcomes. They influence this through enhancing tumor cell proliferation, contributing to matrix assembly, and modulation of signalling pathways like PI3K/AKT, all of which are associated with tumor aggressiveness^72–74^ (also reviewed in ref. ^75^). Further, with the ECM continually being remodelled by ECM-degrading enzymes, degraded ECM components also act as signalling molecules, referred to as matrikines, which influence immune cell function and activity^76^. This highlights a new dimension and a potentially more complex role of the ECM in immunomodulation and underscores the novelty of our findings. Exploring additional influences the ECM may have in the TME, it has been established that the interaction between ECM components (e.g., laminin [LAM]^77,78^, vitronectin [VIT]^50,51^, fibronectin [FN1]^50^) with integrins upregulate BCL2 expression and modulate susceptibility to cell death mechanisms. These interactions have profound implications for current glioma therapies, particularly for small-molecule apoptosis inducers (e.g., BH3 mimetics). Further investigation into this relationship will provide insights into enhancing immunotherapy in HGG. Moving forward, it is critical to consider the ECM not just as a static scaffold but as a dynamic regulator within the tumor microenvironment.

Moreover, ECM composition may directly influence CAR T cell-mediated cytotoxicity. Tumor ECM can act to either physically restrict CAR T cell infiltration or modulate target cell susceptibility to immune-mediated killing. Understanding these interactions will be critical for optimizing next-generation CAR T cell therapies. The role of the ECM as a potential barrier to therapy was previously highlighted by O’Rourke and colleagues and other researchers, who observed limited infiltration of EGFRvIII-directed CAR T cells at the tumor border in glioblastoma^79^. Further, research by Dotti and colleagues on ECM-modifying CAR T cells demonstrated that, upon in vitro expansion, T cells downregulated the ECM-degrading enzyme heparanase^80^. By engineering GD2-directed CAR T cells to co-express heparanase, they achieved enhanced infiltration and anti-tumor activity in a neuroblastoma model^80^. Expanding on this work, future studies could focus on ECM-degrading or modifying enzymes targeting the specific ECM components identified here, thereby improving therapeutic delivery in HGG and other solid tumors.

Beyond enhancing T cell infiltration, targeting ECM-associated proteins directly with CAR T cells represents an exciting therapeutic avenue. Our proteomic data, alongside emerging immunotherapy studies, support the feasibility of ECM molecules as viable CAR T cell targets, with CSPG4^24^, BCAN^27^, TNC^23^, COL11A1^23^, GPC^19,22^ and others already under investigation in preclinical settings. The patient-specific variability in ECM composition across tumors, as reported by Hoogstrate et al.^15^, further suggests that personalized ECM-targeted therapies could significantly improve treatment outcomes, expanding the immunotherapeutic repertoire beyond standard tumor-associated antigen-based CAR T cell therapies. Of note it would be of interest to investigate the effect of ECM-targeted CARs, such as GPC2 CAR T cells, on ECM composition. Though it was not explored here it is known that ECM remodeling enzymes such as heparanase, which cleaves heparan sulfate glycosaminoglycans, is not only overexpressed in a number of tumors but also contributes to tumorigenesis and could also be expressed by activated T cells^47,81–83^. Consequently, ECM-associated targets, like GPC2, may undergo modification on the cell surface. Furthermore, ECM-redirected CARs could preferentially engage ECM components within the TME, rather than canonical cell surface antigens. Whether this interaction would enhance or impair CAR T cell functionality, or be harnessed as a new strategy for CAR T cells, remains an open question that warrants systematic investigation in future studies. In addition, as CAR T cells could facilitate ECM degradation, it is tempting to speculate that ECM targeting might increase immune cell infiltration, also warranting further study.

Despite prior challenges in clinical trials targeting ECM molecules, our study underscores the importance of understanding ECM heterogeneity to enhance the success of future ECM-targeted therapies. Furthermore, our comparison of in vitro glioma models versus primary tumors highlights a critical limitation; in vitro models often fail to accurately replicate ECM composition, emphasizing the need for more physiologically relevant preclinical systems.

Our findings establish the ECM as a key regulator of tumor progression, immune infiltration, and therapy resistance in gliomas. The diverse ECM composition observed across gliomas, coupled with its influence on T cell exclusion and therapy response, underscores its therapeutic relevance. Future efforts should focus on: Developing ECM-targeted CAR T cell therapies, leveraging CSPG4, BCAN, and GPC2 as established ECM antigens; incorporating ECM-modifying enzymes (e.g., heparanase) into CAR T cell constructs to enhance tumor penetration; and investigating ECM-immune interactions to identify strategies that convert gliomas from immune-cold to immune-hot tumors.

By integrating multi-omic datasets, computational target prioritization, and ECM-focused immunotherapies, we can develop next-generation strategies that effectively overcome glioma immune evasion and improve therapeutic outcomes.

## Methods

### Cell culture

The human H4, SW1088, U118 and A172 cell lines were obtained from the American Type Culture Collection (ATCC). The U87 cell line was kindly provided by Rodney Luwor (Royal Melbourne Hospital, The University of Melbourne). The U251 cell lines were kindly provided by Francine Ke (WEHI) and HEK293T cells were also obtained from within WEHI. STR analysis confirmed the identity of all cell lines used in this study. Cell lines were maintained at 37 °C, 5% CO_2_ in RPMI-1640 medium (WEHI) supplemented with 10% fetal calf serum (FCS, Bovogen), 2 mM glutamine (Gibco), 100 U/mL penicillin, and 100 µg/mL streptomycin (Gibco).

Previous nomenclature was used for the patient-derived K27M Diffuse Midline Glioma (DMG) models used in this study including SUDIPG-4, -13, -17, -19, -21, -25, -27, -33, -35, -36, which were a kind gift from Professor Michelle Monje (Stanford University School of Medicine)^84^. These cells were maintained in media at a ratio of DMEM/F-12 (Invitrogen) and Neurobasal-A Medium (Invitrogen) supplemented with 10 mM HEPES (Gibco), 1 mM sodium pyruvate (Gibco), 0.1 mM Minimal Essential Media (MEM) non-essential amino acids (NEAA, Gibco), 2 mM GlutaMAX-I (Gibco), antibiotic-antimycotic (containing 100 units/mL of penicillin, 100 ug/mL of streptomycin, 0.25 ug/mL of Gibco Amphotericin B (Gibco), B27 supplement (Invitrogen), 20 ng/mL epidermal growth factor (EGF, Shenandoah Biotech), 20 ng/mL fibroblast growth factor (FGF, Shenandoah Biotech), 10 ng/mL platelet-derived growth factor (PDGF)-AA, (Shenandoah Biotech), 10 ng/mL PDGF-BB (Shenandoah Biotech), and 2 µg/mL Heparin (StemCell Technologies). All cells were verified as Mycoplasma negative by polymerase chain reaction (PCR) analysis at the WEHI internal facility and were cultured and maintained at 37 °C in 5% CO_2_.

Primary human peripheral blood mononuclear cells (PBMCs) were obtained from the Australian Red Cross (Agreement #23-06VIC-16). Human T cells isolated from the PBMCs were maintained in RPMI (WEHI) enriched with 10% FCS, 1 mM sodium pyruvate (Gibco), 2 mM GlutaMAX-I (Gibco), 0.1 mM NEAA (Gibco), 50 µM Beta-mercaptoethanol (Sigma), 100 U/mL penicillin (Gibco), and 100 µg/mL streptomycin (Gibco), and 50 IU/mL rhIL-2 (Peprotech, #200–02).

### Ethics

This study was conducted in accordance with the Declaration of Helsinki. The use of primary human brain tissue was approved by the Walter and Eliza Hall Institute (WEHI) Human Research Ethics Committee (Projects 21/21; 2009.016 and G17.10). Ethics approval was obtained from the following institutions: the Australian and New Zealand Children’s Hematology/Oncology Group (ANZCHOG) Biobanking Network, including the Royal Children’s Hospital (Melbourne, Victoria), and the Royal Melbourne Hospital (Melbourne, Victoria). Pediatric tumor samples and coded data were provided by the Children’s Cancer Centre Tissue Bank at the Murdoch Children’s Research Institute and The Royal Children’s Hospital (www.mcri.edu.au/childrenscancercentretissuebank). Written informed consent was obtained from all participants or their legal guardians.

### Primary human high-grade glioma samples

Tumors were collected from patients undergoing resection either at the Royal Melbourne Hospital (Melbourne, Australia) or the Royal Children’s Hospital (Melbourne, Australia). The pediatric HGG (pHGG) cohort consisted of six primary tumors (*n* = 6 tissue blocks), including 3 glioblastomas (pGBM) and 3 DMGs, originally classified as diffuse intrinsic pontine gliomas (pDIPG). The patient ages in this group ranged from 5 to 11 years, including five males and one female (Supplementary Table 1). The adult HGG (aHGG) cohort included a diverse group of thirty primary tumors (n = 40 tissue blocks), comprising 18 cases of glioblastoma (aGBM), 3 high-grade gliomas (aHGG), 6 astrocytomas (AST), 2 oligodendrogliomas (OLGD), and 1 haemangioblastoma (HMGB). The average age of this cohort was 54 years, with 20 male and 9 female patients (1 patient of unknown sex) (Supplementary Table 5).

Both adult and pediatric tumor samples were processed as formalin-fixed, paraffin-embedded (FFPE) blocks until further use.

### Immunohistochemistry and staining of primary patient high-grade glioma samples and quantification using QuPath

Formalin-fixed paraffin-embedded (FFPE) tissue blocks (described above) were subject to serial sectioning, dewaxed, and heat-mediated antigen retrieval using a microwave in 0.01 M sodium citrate buffer (pH 6.0) was performed on all sections. After cooling, sections were washed, and endogenous peroxidases were quenched using 1% hydrogen peroxide for 10 min. Nonspecific antibody binding was blocked using 10% normal goat serum from Vector Laboratories (Burlingame). The serial sections from each tumor were stained with Haematoxylin and Eosin (H&E), Alcian Blue (AB) (glycosaminoglycans), Massons Trichome (MT) (collagen) as well as *a*CD4 (Abcam, AB133616, clone: EPR6855) and *a*CD8 (Abcam, AB101500, lot:1058464-41, clone: SP16) antibodies. Slides were mounted using DPX mountant (Sigma-Aldrich) and scanned using a SLIDEVIEW VS200 Slide Scanner at 20X (Olympus, Tokyo, Japan). Image files for imported into QuPath V.0.4.3 for analysis. Whole slides were analyzed for regions of interest and annotated for regions of interest (to identify areas with tumor and areas of high and low AB and MT staining).

### Histology scoring

All slides (from above) were blindly scored by an anatomical pathologist for extent and intensity of staining. Briefly, for each sample the H&E-stained slide was assessed first to ensure that tumor was present. Then, each AB, MT and *a*CD4/8+ slide was scored semi-quantitatively on a scale from 1 to 4 (H-Score). For scoring purposes, 1 is considered negligible staining, and 4 is considered strong and widespread staining. Scores of 2 and 3 were then allocated designations ‘low staining’ and ‘moderate staining’, respectively.

Each slide was reviewed 3 times. In the first review, slides with negligible staining were allocated with a score of 1, and slides with the greatest staining were allocated a 4. The remaining slides were allocated with intermediary scores of 2 or 3. Slides were then reviewed twice more to ensure consistency and accuracy of scoring across the adult and pediatric cohorts. No necrotic tissue was identified in any samples.

A composite ‘ECM Score’ was also derived for the purposes of data analysis. Here, the ECM score is the sum of the Alcian Blue H-Score and the Massons Trichome H-Score for each individual tumor sample. This score gives us insight into the extent of ECM deposition as it allows us to simultaneously quantify both glycosaminoglycan (Alcian Blue) and collagen (Massons Trichome) deposition.

### Cell Surfaceome Cell Preparation and Data

Cell surface proteomics was performed on each sample in quadruplicate using an amino-oxy-biotin labeling technique, previously described^85^. Briefly, fresh tumor samples or cell lines were mechanically dissociated into single-cell suspension and exposed surface sialic acid residues were biotinylated with sodium meta-periodate, aniline, and amino-oxy-biotin (Biotium). Nuclei were removed by centrifugation and biotinylated proteins enriched with streptavidin-agarose (Pierce). Proteins were digested off the beads with Trypsin Gold and peptides were collected by centrifugation. Tryptic fractions were analyzed for mass spectrometry analysis with an Impact II operated in a data-dependent mode. Raw files were analyzed using MaxQuant. The database search was performed using the UniProt Homo Sapiens database and the relative abundance of proteins was determined using intensity based absolute quantitation (iBAQ) (total precursor intensities divided by the theoretically observable number of peptides).

### Extracellular matrix RNAseq data from The Cancer Genome Atlas (TCGA)

The RNAseq results published here are in whole or part based upon data generated by the TCGA Research Network: https://www.cancer.gov/tcga. RNAseq data for all the extracellular matrix components listed in Supplementary Table 2 and Supplementary Table 3 as extracted from TCGA data sets and plotted to compare expression in adult and pediatric High-Grade Gliomas. The adult data sets interrogated were the Glioblastoma, TCGA PanCancer Atlas (*n* = 160 tumors) (Supplementary Data 1). The pediatric data set interrogated was the Pediatric Brain Tumor Atlas, PBTA Provisional (*n* = 1945 tumors) (Supplementary Data 2).

### ImmunoTar analysis

Cell surface proteomic data were analyzed using ImmunoTar, a computational workflow which enriches user-provided protein abundance data with relevant publicly available bioinformatic datasets to generate a genes-by-feature matrix (publicly available, 25). Subsequent analysis of this matrix by ImmunoTar ranks the supplied data according to a prediction of each protein’s suitability as an immunotherapeutic target.

Briefly, cell surface proteomic datasets were used as the primary input for ImmunoTar analysis. To enrich feature representation, ImmunoTar incorporated multiple publicly available datasets, including GTEx for normal tissue expression profiles, UniProt for subcellular localization and protein function, DepMap for gene dependency scores across cancer cell lines, Gene Ontology for functional annotation, and FDA Pediatric Molecular Target lists to highlight relevance in pediatric oncology. Additional data on surface protein likelihood were sourced from CIRFESS and the Compartments databases. A comprehensive gene-by-feature matrix was generated, where each row represented a gene and each column corresponded to a feature relevant to immunotherapy suitability (e.g., tumor specificity, surface localization, essentiality). All features were scaled to a common range (typically 0 to 1), and missing values were handled through imputation using k-nearest neighbor or median substitution. Optional nonlinear transformations were applied to accentuate biologically meaningful differences in feature values. Each gene was assigned a composite ImmunoTar score computed as a weighted sum of its individual features.

For adult-derived proteomic data, the default enrichment settings of ImmunoTar were used. For pediatric-derived proteomic data, enrichment settings were restricted to pediatric-relevant enrichment datasets where possible.

### Genetic constructs

The CAR construct used in this study was modified from a lentiviral transfer vector pRRLSIN-WPRE-GFP (Addgene #12252) wherein WPRE was substituted with an EF-1α minimal promoter and GFP with a CAR. The second-generation CAR comprised of a single-chain variable fragment (scFv) with a G4S linker, a CD8 hinge, a CD28 transmembrane domain, a CD28 costimulatory domain and a CD3ζ signalling tail. The CAR construct also contains a truncated, non-signalling EGFRvIII cell-surface protein (nsEGFRvIII) sequence. The construct was previously generated in our lab using Gibson Assembly cloning of a gene block designed to contain amino acids 1–26, G mutation and amino acids 298–671 of EGFR, and hence, is truncated intracellularly and a non-signalling variant. The nsEGFRvIII can be bound via cetuximab antibody which facilities detection of ECM- degrading enzyme vectors to allow determination of transduction efficiency. The Glypican-2 (GPC2) CAR consisted of anti-GPC2 scFv (clone 27 derived from previous studies^19^) and the control T cells were subjected to the transduction process without a transfer plasmid. The CD19 CAR consisted of scFv clone FMC63, with amino acid sequence (from addgene plasmid #135993) and cross referenced against patented claims before being cloned into the CD28/CD3ζ second-generation CAR backbone.

### T cell transduction

Lentivirus was produced via transfection of HEK293T cells using the plasmids: pMD2G-VSVg (Addgene #12259), pMDLg/pRRE (Addgene #12251), pRSVREV (Addgene #12253), and CAR transfer vector pRRLSIN-EF1α-CAR using FuGENE-6 (Promega) as per the manufacturer’s protocol. Human CD8^+^ T cells were selected from healthy donor PBMCs using CD8^+^ positive selection kits (EasySep, StemCell Technologies). Selected T cells were activated with αCD3/CD28 Dynabeads (Life Technologies) for 48 h. Two rounds of transduction of the human T cells were performed on consecutive days (48 and 72 h post-activation) on retronectin-coated plates (Takara Bio), which were spinoculated for 1 h at 1000 G and 22 °C.

### Cytotoxicity assay

Target cells were seeded in triplicate in 96 flat-bottom plates before co-culture with human CAR T cells in the presence of 50 µM propidium iodide (PI, Calbiochem). PI uptake into target cells was measured as a surrogate for cell death, and images were captured every hour for 72 h by the IncuCyte (Sartorius, Models S3, SX5). Results were analyzed using IncuCyte software (Sartorius, V.2021A).

## Supplementary information

Supplementary Tables and Figures_250507

Supplementary Data 1

Supplementary Data 2

## Data Availability

The datasets generated and analysed during this study are available from the corresponding author upon reasonable request.
