# Supplementary material for: Defining the extracellular matrix for targeted immunotherapy in adult and pediatric brain cancer
Source: NPJ Precis Oncol. 2025 Jun 14;9:184. doi: 10.1038/s41698-025-00956-z (PMC12167366; doi:10.1038/s41698-025-00956-z)
Supplement: Supplementary file 1 — Supplementary Tables and Figures_250507 [file 41698_2025_956_MOESM1_ESM.pdf]

Age	Sex	Diagnosis	ID	Grade	H3K27M	GFAP	OLIG2	INI-1	p53	Ki67
5	F	DIPG	DIPG 1*	4	mutated	positive	positive	-	wildtype	high
5	M	DIPG	DIPG 2*	4	mutated	-	-	-	-	-
5	M	DIPG	DIPG 3	4	mutated	positive	-	mutated	wildtype	50%
4	M	GBM	GBM 1	4	-	-	-	-	-	-
11	M	GBM	GBM 2	-	-	positive	-	-	-	20%
7	M	GBM	GBM 3	4	-	positive	-	retained	mutated	-

Supplementary Table 1. Patient Metadata and Diagnosis for Pediatric Tumor Samples used for both Histological and Proteomic Analysis. Patient Metadata for Pediatric Glioblastoma and Diffuse Intrinsic Pontine Glioma Tumor Specimens that contributed tumor samples to this study including age, sex, diagnosis, tumor grade, identifiers as well as genetic status and immunoreactivity of tumors. GBM = Glioblastoma, DIPG = Diffuse Intrinsic Pontine Glioma. (n=6 patients provided n=6 tumors blocks) (* indicates tumors that underwent proteomic analysis).

8

Category	Name	Gene Name	Predominant GAG	UniProt ID	Cellular Localisation	Size (aa)
Heparan Sulfate Proteoglycan (HSPG)	Collagen XVIII	COL18A1	Heparan Sulfate (HS)	P39060	Secreted	1754
Heparan Sulfate Proteoglycan (HSPG)	Glypican	GPC1	Heparan Sulfate (HS)	P35052	Cell Surface GPI Anchored/Secreted	558
Heparan Sulfate Proteoglycan (HSPG)	Glypican	GPC2	Heparan Sulfate (HS)	Q8N158	Cell Surface GPI Anchored/Secreted	579
Heparan Sulfate Proteoglycan (HSPG)	Glypican	GPC3	Heparan Sulfate (HS)	P51654	Cell Surface GPI Anchored	580
Heparan Sulfate Proteoglycan (HSPG)	Glypican	GPC4	Heparan Sulfate (HS)	O75487	Cell Surface GPI Anchored/Secreted	556
Heparan Sulfate Proteoglycan (HSPG)	Glypican	GPC5	Heparan Sulfate (HS)	P78333	Cell Surface GPI Anchored/Secreted	572
Heparan Sulfate Proteoglycan (HSPG)	Glypican	GPC6	Heparan Sulfate (HS)	Q9Y625	Cell Surface GPI Anchored/Secreted	555
Heparan Sulfate Proteoglycan (HSPG)	Perlecan	HSPG2	Heparan Sulfate (HS)	P98160	Secreted/Basement Membrane	4391
Heparan Sulfate Proteoglycan (HSPG)	Syndecan	SDC1	Heparan Sulfate (HS)	P18827	Cell Surface/Single-pass type I membrane protein	310
Heparan Sulfate Proteoglycan (HSPG)	Syndecan	SDC2	Heparan Sulfate (HS)	P34741	Cell Surface/Single-pass type I membrane protein	201
Heparan Sulfate Proteoglycan (HSPG)	Syndecan	SDC3	Heparan Sulfate (HS)	O75056	Cell Surface/Single-pass type I membrane protein	442
Heparan Sulfate Proteoglycan (HSPG)	Syndecan	SDC4	Heparan Sulfate (HS)	P31431	Cell Surface/Single-pass type I membrane protein	198
Heparan Sulfate Proteoglycan (HSPG)	Testican	SPOCK1	Heparan Sulfate (HS)	Q08629	Secreted	439
Heparan Sulfate Proteoglycan (HSPG)	Testican	SPOCK2	Heparan Sulfate (HS)	Q92563	Secreted	424
Heparan Sulfate Proteoglycan (HSPG)	Testican	SPOCK3	Heparan Sulfate (HS)	Q9BQ16	Secreted	436
Heparan Sulfate Proteoglycan (HSPG)	Serglycin	SRGN	Heparan Sulfate (HS)	P10124	Secreted/Golgi	158
Heparan Sulfate Proteoglycan (HSPG)	Agrin	AGRN	Heparan Sulfate (HS)	O00468	Cell Surface/Single-pass type II membrane protein/Secreted	2068
Chondroitin Sulfate Proteoglycan (CSPG)	Brevican	BCAN	Chondroitin Sulfate (CS)	Q96GW7	Cell Surface/Membrane Bound GPI Anchored	911
Chondroitin Sulfate Proteoglycan (CSPG)	Biglycan	BGN	Chondroitin Sulfate (CS)	P21810	Secreted	368
Chondroitin Sulfate Proteoglycan (CSPG)	NG2/CSPG4	CSPG4	Chondroitin Sulfate (CS)	Q6UVK1	Cell Surface/Single-pass type I membrane protein	2322
Chondroitin Sulfate Proteoglycan (CSPG)	Neuroglycan/CSPG5	CSPG5	Chondroitin Sulfate (CS)	O95196	Cell Surface/Single-pass type I membrane protein/Neuronal Synapse Secreted	566
Chondroitin Sulfate Proteoglycan (CSPG)	Necrocan	NCAN	Chondroitin Sulfate (CS)	O14594	Secreted	1321
Chondroitin Sulfate Proteoglycan (CSPG)	Phosphocan	PTPRZ1	Chondroitin Sulfate (CS)	P23471	Cell Surface/Single-pass type I membrane protein	2315
Chondroitin Sulfate Proteoglycan (CSPG)	Betaglycan	TGFBR3	Chondroitin Sulfate (CS)	Q03167	Cell Surface/Single-pass type I membrane protein	851
Chondroitin Sulfate Proteoglycan (CSPG)	Versican	VCAN	Chondroitin Sulfate (CS)	P13611	Secreted/Cell Porjection,Cilium, Photoreceptor	3396
Chondroitin Sulfate Proteoglycan (CSPG)	Collagen XV	COL15A1	Chondroitin Sulfate (CS)/Heparan Sulfate (HS)	P39059	Secreted	1388
Chondroitin Sulfate Proteoglycan (CSPG)	Aggrecan	ACAN	Chondroitin Sulfate (CS)/Keratan Sulfate (HS)	P16112	Secreted	2530
Keratan Sulfate Proteoglycan (KSPG)	Asporin	ASPN	Keratan Sulfate (HS)	Q9BXN1	Secreted	380
Keratan Sulfate Proteoglycan (KSPG)	Osteoglycin/Mimecan	OGN	Keratan Sulfate (HS)	P20774	Secreted	298
Keratan Sulfate Proteoglycan (KSPG)	Fibromodulin	FMOD	Keratan Sulfate (HS)	Q06828	Secreted	376
Keratan Sulfate Proteoglycan (KSPG)	Lumican	LUM	Keratan Sulfate (HS)	P51884	Secreted	338
Keratan Sulfate Proteoglycan (KSPG)	Prolargin	PRELP	Keratan Sulfate (HS)	P51888	Secreted	382
Keratan Sulfate Proteoglycan (KSPG)	Keratocan	KERA	Keratan Sulfate (HS)	O60938	Secreted	352
Dermatan Sulfate Proteoglycan (DSPG)	Decorin	DCN	Dermatan Sulfate (DS)	P07585	Secreted	359
Other	Epiphycan	EPYC	Dermatan Sulfate (DS)/Chondroitin Sulfate (CS)	Q99645	Secreted	322
Other	Extracellular matrix protein 2	ECM2	-	O94769	Secreted	699
Other	Osteoadherin	OMD	-	Q99983	Secreted	421
Other	Chondroadherin	CHAD	-	O15335	Secreted	359
Other	Nyctalopin	NYX	-	Q9GZU5	Secreted	476
Other	Tsukushi	TSKU	-	Q8WUA8	Secreted	353
Other	Podocan	PODN	-	Q7Z5L7	Secreted/Cytoplasm	613

Supplementary Table 2. List of Extracellular Matrix Proteoglycans Interrogated in this Study. Information including family, name, gene, associated glycosaminoglycan (GAG), UniProt ID, localization and size (amino acids) of the proteoglycans investigated as a part of the characterization of the extracellular matrix of adult and pediatric high-grade gliomas.

Category	Name	Gene Name	UniProt ID	Cellular Localisation	Size (aa)
Fibrous/Glycoprotein	Collagen II	COL2A1	P02458	Secreted	1487
Fibrous/Glycoprotein	Collagen III	COL3A1	P02461	Secreted	1466
Fibrous/Glycoprotein	Collagen IV	COL4A3	Q01955	Secreted/Basement Membrane	1670
Fibrous/Glycoprotein	Collagen IV	COL4A4	P53420	Secreted/Basement Membrane	1690
Fibrous/Glycoprotein	Collagen IV	COL4A5	P29400	Secreted/Basement Membrane	1685
Fibrous/Glycoprotein	Collagen IV	COL4A6	Q14031	Secreted/Basement Membrane	1691
Fibrous/Glycoprotein	Collagen VI	COL6A1	P12109	Secreted	1028
Fibrous/Glycoprotein	Collagen VI	COL6A2	P12110	Secreted (Recruited on membranes by CSPG4)	1091
Fibrous/Glycoprotein	Collagen VI	COL6A3	P12111	Secreted	3177
Fibrous/Glycoprotein	Collagen VII	COL7A1	Q02388	Secreted/Basement Membrane	2944
Fibrous/Glycoprotein	Collagen VIII	COL8A2	P25067	Secreted/Basement Membrane	703
Fibrous/Glycoprotein	Collagen XI	COL11A1	P12107	Secreted	1806
Fibrous/Glycoprotein	Collagen XI	COL11A2	P13942	Secreted	1736
Fibrous/Glycoprotein	Collagen XIV	COL141A	Q05707	Secreted	1796
Fibrous/Glycoprotein	Collagen XVII	COL17A1	Q9UMD9	Cell Surface/Single-pass type II membrane protein (hemidesmosome)	1497
Fibrous/Glycoprotein	Collagen I	COL1A1	P02452	Secreted	1464
Fibrous/Glycoprotein	Collagen I	COL1A2	P08123	Secreted	1366
Fibrous/Glycoprotein	Elastin	ELN	P15502	Secreted	786
Fibrous/Glycoprotein	Fibronectin	FN1	P02751	Secreted	2477
Fibrous/Glycoprotein	Entactin	NID1	P14543	Secreted/Basement Membrane	1247
Fibrous/Glycoprotein	Plasminogen	PLG	P00747	Secreted	810
Fibrous/Glycoprotein	Thrombospondin-2	THBS2	P35442	Basement Membrane	1172
Fibrous/Glycoprotein	Tenascin C	TNC	P24821	Secreted	2201
Fibrous/Glycoprotein	Tenascin R	TNR	Q92752	Secreted	1358
Fibrous/Glycoprotein	Tenascin X	TNXB	P22105	Secreted	4244
Fibrous/Glycoprotein	Vitronectin	VTN	P04004	Secreted	478

Supplementary Table 3. List of Extracellular Matrix Collagen and Glycoproteins Interrogated in this Study. Information including category, name, gene, associated glycosaminoglycan (GAG), UniProt ID, localization and size (amino acids) of the collagen isotypes and glycoprotein investigated as a part of the characterization of the extracellular matrix of adult and pediatric high-grade gliomas.

Diagnosis	Identifier	Type	Surgery	Grade	Patient Age	Sex	Ki67	IDH Status	ATRX	p53	1p/19 co-deleted	TERT	EGFR
GA	CS-GA1	Primary	Debulking	II	50	F	-	mutated	-	-	-	-	-
OLGD	CS-OLGD1	Primary	Debulking	II	24	M	<4%	mutated	unmutated	mutated	yes	-	-
OLGD	CS-OLGD2	Primary	Debulking	III	46	M	-	mutated	-	-	yes	mutated	not amplified
GBM	CS-GBM1	Primary	Biopsy	IV	58	M	<5%	wild type	unmutated	mutated	-	-	-
GBM	CS-GBM2	Primary	Debulking	IV	43	F	-	wild type	-	-	-	mutated	not amplified
GBM	CS-GBM3	Primary	Debulking	IV	77	F	20%	wild type	unmutated	mutated	-	-	-
GBM	CS-GBM4	Primary	Debulking	IV	50	M	10%	wild type	-	-	-	mutated	not amplified
GSM	CS-GSM1	Primary	Debulking	IV	45	M	<5%	wild type	unmutated	mutated	-	-	-

Supplementary Table 4. Patient Metadata and Diagnosis for Adult Brain Cancer Proteomic Samples. Metadata of 10 adults diagnosed with glioblastoma (GBM), gemistocytic astrocytoma (GA) or Oligodendroglioma (OLGD) that contributed tumor samples for proteomic analysis in this study including age, sex, diagnosis, tumor grade, identifiers as well as genetic status and immunoreactivity of tumors.

Age	Sex	Diagnosis	ID	Grade	IDH Status	GFAP	OLIG2	IDH1 R132H	ATRX	p53	p16 CDKN2A	Ki67	MGMT Promotor
75	F	GBM	GBM 1	4	wildtype	positive	positive	wildtype	unmutated	wildtype	negative	0.5	unmethylated
63	M	GBM	GBM 2	4	wildtype	positive	positive	wildtype	unmutated	mutated	positive	0.3	methylated
35	M	GBM	GBM 3	4	wildtype	positive	positive	wildtype	unmutated	wildtype	negative	0.15	methylated
39	M	GBM	GBM 4	4	wildtype	positive	positive	wildtype	unmutated	wildtype	negative	0.5	-
65	M	GBM	GBM 5	4	wildtype	positive	negative	wildtype	unmutated	mutated	positive	0.6	unmethylated
-	-	GBM	GBM 6	-	-	-	-	-	-	-	-	-	-
60	M	GBM	GBM 7	4	wildtype	positive	positive	wildtype	unmutated	mutated	negative	20-30%	methylated
24	M	GBM	GBM 8	4	negative	positive	positive	wildtype	unmutated	wildtype	positive	0.4	methylated
42	M	GBM	GBM 9	4	wildtype	positive	positive	wildtype	unmutated	wildtype	negative	0.2	methylated
78	M	GBM	GBM 10	4	wildtype	positive	patchy	wildtype	unmutated	wildtype	positive	25-30%	methylated
63	M	GBM	GBM 11	4	wildtype	positive	positive	wildtype	unmutated	wildtype	negative	0.6	methylated
55	F	GBM	GBM 12	4	wildtype	positive	positive	wildtype	unmutated	wildtype	negative	30-40%	unmethylated
48	M	GBM	GBM 13	4	wildtype	positive	positive	wildtype	unmutated	wildtype	wildtype	0.3	unmethylated
82	F	GBM	GBM 14	4	wildtype	positive	positive	wildtype	unmutated	wildtype	negative	30-40%	-
68	M	GBM	GBM 15	4	wildtype	Positive	positive	wildtype	unmutated	mutated	positive	0.6	-
73	M	GBM	GBM 16	4	wildtype	positive	positive	wildtype	unmutated	wildtype	negative	0.4	methylated
44	M	GBM	GBM 17	4	wildtype	positive	positive	wildtype	mutated	mutated	positive	50%.	unmethylated
62	M	GBM	GBM 18	4	wildtype	positive	positive	wildtype	unmutated	wildtype	negative	0.6	methylated
40	M	AST	AST 1	4	mutated	positive	positive	mutated	mutated	equivocal	positive	0.4	methylated
35	M	AST	AST 2	4	mutated	positive	positive	wildtype	mutated	mutated	positive	0.2	
45	F	AST	AST 3	1	wildtype	positive	positive	wildtype	unmutated	wildtype	positive	<1%	-
54	M	AST	AST 4	3	mutated	positive	positive	mutated	mutated	mutated	patchy staining	0.05	-
46	F	AST	AST 5	4	mutated	positive	positive	mutated	mutated	mutated	negative	0.15	-
27	M	AST	AST 6	3	mutated	positive	positive	mutated	mutated	mutated	positive	0.3	-
51	M	HGG	HGG 1	4	wildtype	positive	positive	wildtype	unmutated	wildtype	negative	0.3	methylated
68	F	HGG	HGG 2	4	wildtype	positive	positive	wildtype	unmutated	wildtype	equivocal	0.2	methylated
70	F	HGG	HGG 3	4	wildtype	positive	positive	wildtype	unmutated	equivocal	negative	0.4	methylated
47	M	OLGD	OLGD 1	3	mutant	-	-	-	-	-	-	-	-
81	F	OLGD	OLGD 2	3	mutated	positive	positive	mutated	unmutated	wildtype	positive	20-25%	-
31	F	HMGB	HMGB 1	-	-	-	-	-	-	-	-	-	-

Supplementary Table 5. Patient Data for Adult Brain Cancer Tumor Specimens that underwent Histological Analysis. Metadata of a 30 adult cohort that contributed tumor samples to this study including age, sex, diagnosis, tumor grade, identifiers as well as genetic status and immunoreactivity of tumors; Diagnosis are as follows GBM = Glioblastoma, HGG = High Grade Glioma, AST = Astrocytoma, OLGD = oligodendroglioma, HMGB = Haemangioblastoma (n=30 patient samples provided n=40 tumors blocks, some samples were divided into more than one tumor block for analysis).

Sample ID	Alcian Blue (H-Score)	Massons Trichome (H-Score)	α-CD4^{+}/α-CD8^{+} (H-Score)
GBM 6 A	3	4	4
GBM 6 B	3	4	4
GBM 6 C	3	4	4
GBM 1 A	3	4	4
GBM 1 B	3	3	4
GBM 2 A	3	3	4
GBM 2 B	3	3	4
GBM 2 C	2	3	4
GBM 2 D	3	2	4
GBM 3	2	2	4
GBM 4	2	2	4
GBM 12	3	3	3
GBM 5	3	3	3
GBM 10 A	3	3	3
GBM 10 B	3	3	3
GBM 7	2	3	3
GBM 14 A	3	2	3
GBM 15	2	2	3
GBM 11 A	3	1	3
GBM 11 B	3	1	3
HGG 1	2	1	3
AST 4	2	1	3
HGG 3	2	1	3
HGG 2 A	4	3	2
HGG 2 B	2	3	2
HGG 2 C	3	2	2
OLGD 1 A	2	2	2
OLGD 1 B	2	1	2
AST 2	2	1	2
AST 5	2	1	2
AST 6	2	1	2
GBM 16	2	1	2
GBM 17	2	1	2
GBM 18	2	1	2
OLGD 1 E	2	1	2
OLGD 2	2	1	2
AST 1	2	1	2
GBM 13	1	1	2
GBM 8	3	1	1
GBM 9	2	1	1

Supplementary Table 6. Patho-Histological Scoring (H-Score) of Alcian Blue, Massons Trichome and dual a-CD4^{+}/a-CD8^{+} Staining in Adult Primary Brain Cancers. Raw patho-histological score (H-Score) of all 40 primary adult brain cancer samples demonstrating the relationship between alcian blue, masons trichome and dual a-CD4^{+}/a-CD8^{+} staining between tumor samples.

Sample ID	Alcian Blue (H-Score)	Massons Trichome (H-Score)	α-CD4$^+$/α-CD8$^+$ (H-Score)
GBM 3	4	2	2
GBM 2	3	2	2
GBM 1	3	2	2
DIPG 1	3	2	2
DIPG 3	3	2	2
DIPG 2	2	1	1

Supplementary Table 7. Patho-Histological Scoring (H-Score) of Alcian Blue, Massons Trichome and dual a-CD4$^+$/a-CD8$^+$ Staining in Pediatric High-Grade Gliomas. Raw patho-histological (H-Score) score of all 6 primary pediatric high-grade gliomas demonstrating the relationship between alcian blue, masons trichome and dual a-CD4$^+$/a-CD8$^+$ staining between tumor samples.

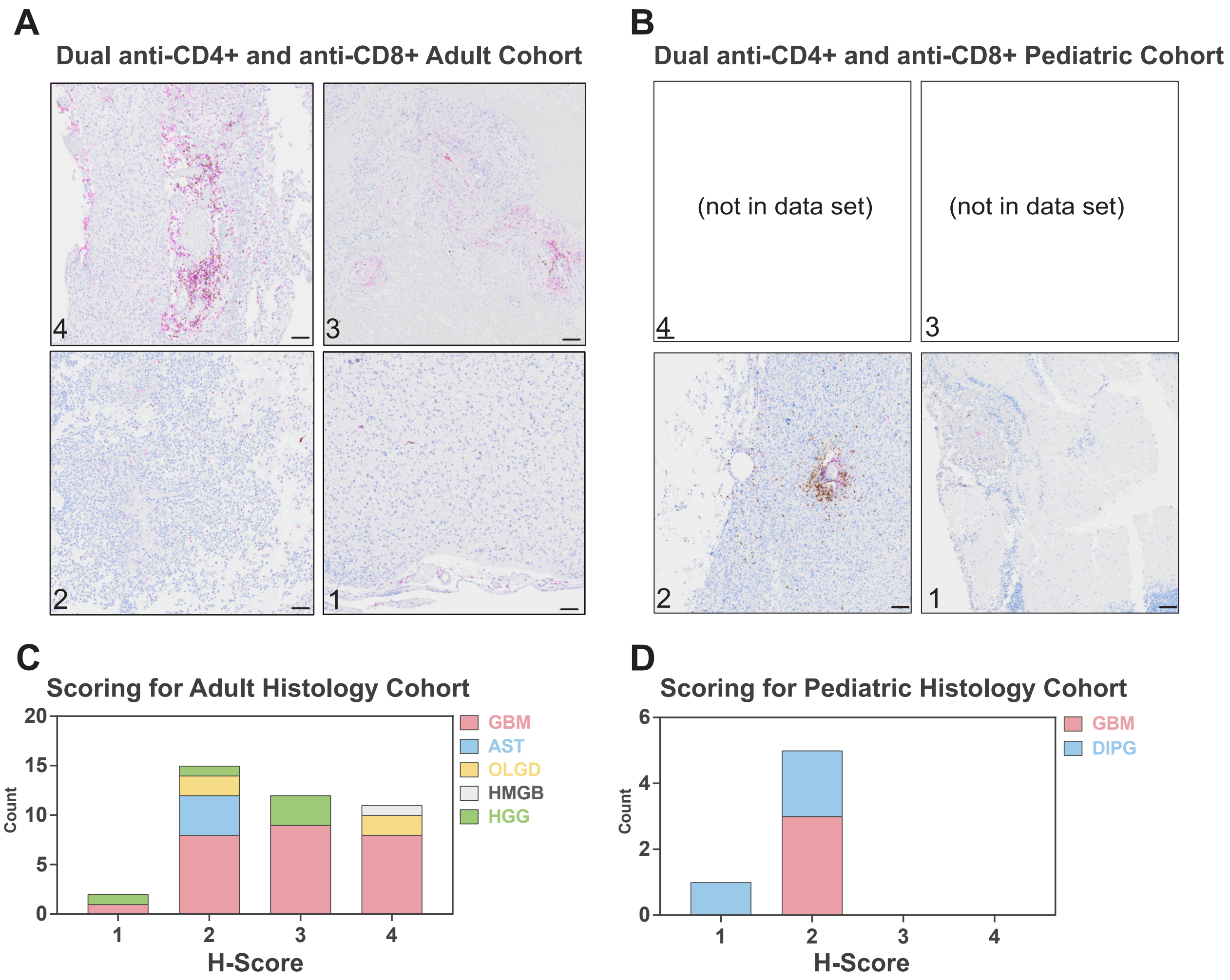

Supplementary Figure 1. Endogenous T cell ($CD4^+/CD8^+$) Staining in Adult and Pediatric Brain Cancers. Histology was performed on 40 primary Adult High Grade Glioma tumor samples [across a 30-patient cohort] and 6 rare primary pediatric high-grade gliomas and scored by an anatomical pathologist. Staining was performed on serial cut tumor sections, here we show examples of $CD4^+$ (magenta) and $CD8^+$ (brown) staining at all detected scoring levels in **(A)** adult brain cancers ranging from tumors that scored a 4, 3, 2 or 1 and **(B)** pediatric high-grade gliomas ranging from tumors that scored a 2 or 1 (no score 4 or score 3 was identified in this cohort) (scale bar = 100 μm). Quantification of the scoring of $CD4^+$ and $CD8^+$ staining for adult pediatric cohorts is shown in **(C)** and **(D)**, respectively.

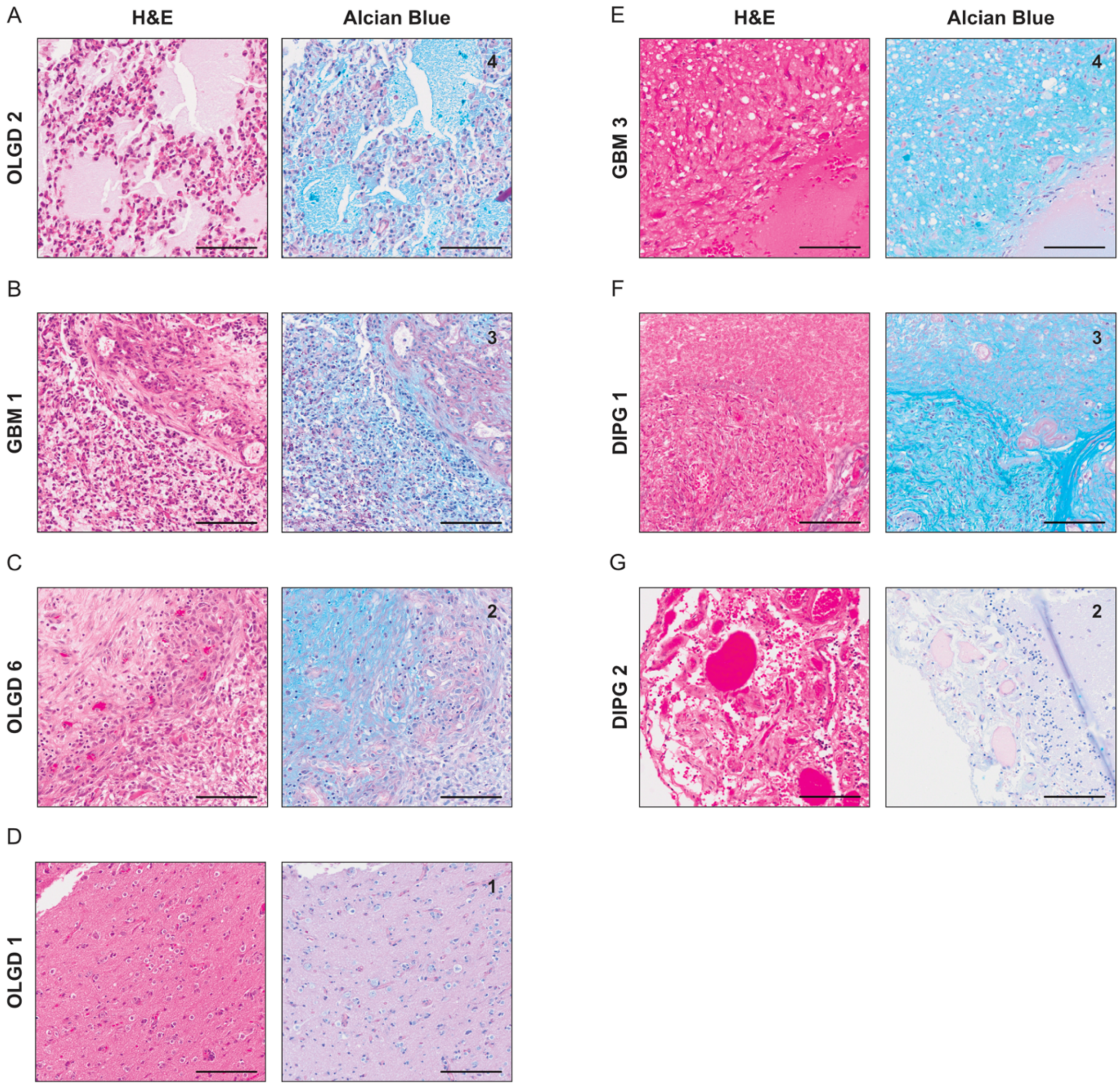

Supplementary Figure 2. Presence of Glycosaminoglycans in the ECM of Adult and Pediatric High Grade Glioma. Histology was performed on 40 primary Adult High Grade Glioma tumors samples [across a 30-patient cohort] and scored by an anatomical pathologist. Staining was performed on serial tumor sections, here we show Alcian Blue (AB) staining in adult high-grade gliomas ranging from tumors that scored a **(A)** 4 **(B)** 3 **(C)** **2** and **(D)** 1 (scale bar = 100 μm). Alcian blue staining was also performed on 6 primary pediatric high-grade gliomas and scored by an anatomical pathologist. Here we show Alcian Blue (AB) staining in pediatric high-grade gliomas ranging from tumors that scored a **(E)** 4 **(F)** 3 and **(G)** 2 (scale bar = 200 μm).

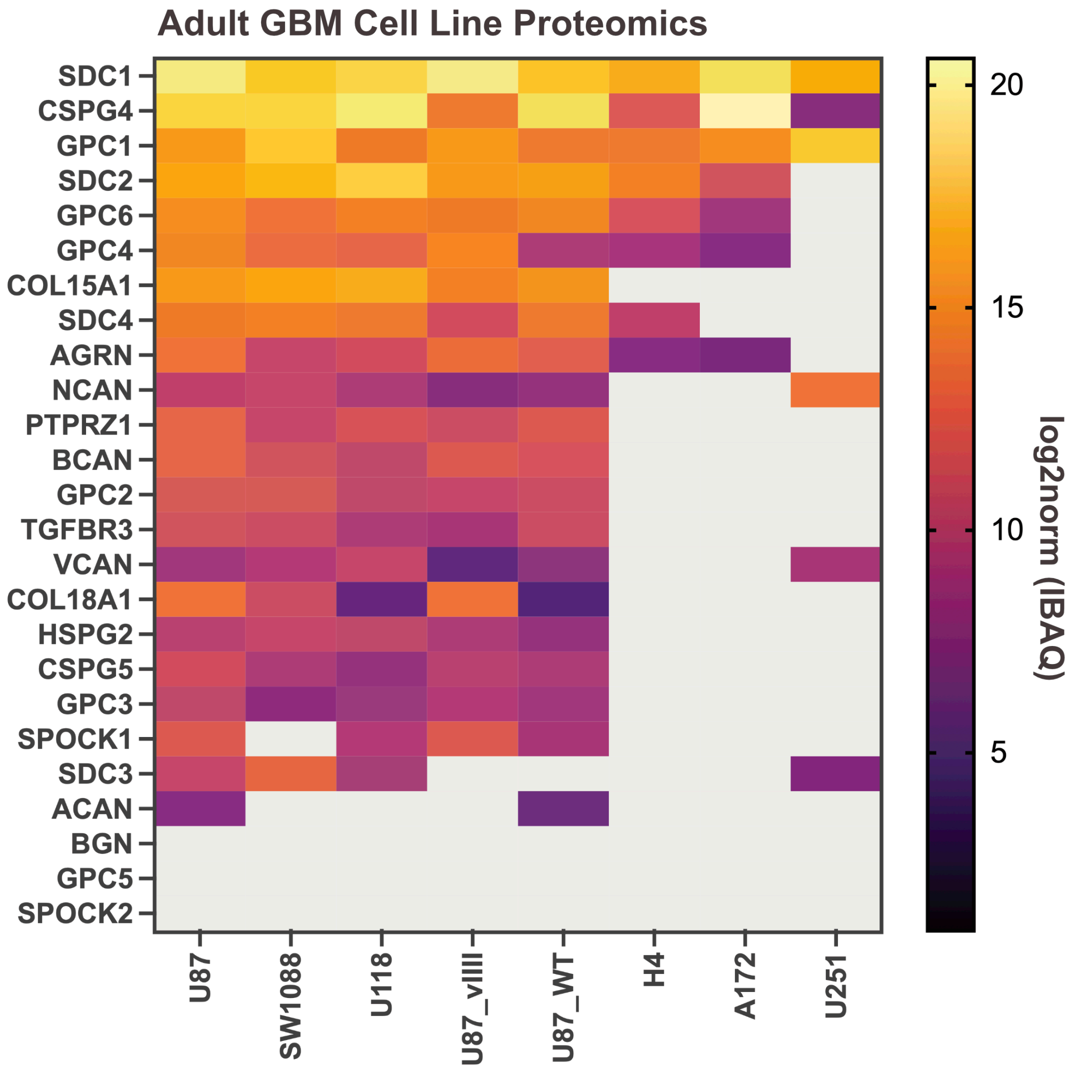

Supplementary Figure 3. Presence of Heparan and Chondroitin Sulfate Proteoglycans in Glioblastoma Model Cell Lines. Heat map showing cell surface proteomic data, as detected by mass spectrometry, of 8 glioblastoma cell lines showing the presence of heparan and chondroitin sulfate proteoglycans detected at the cell surface (grey indicates lack of detection).

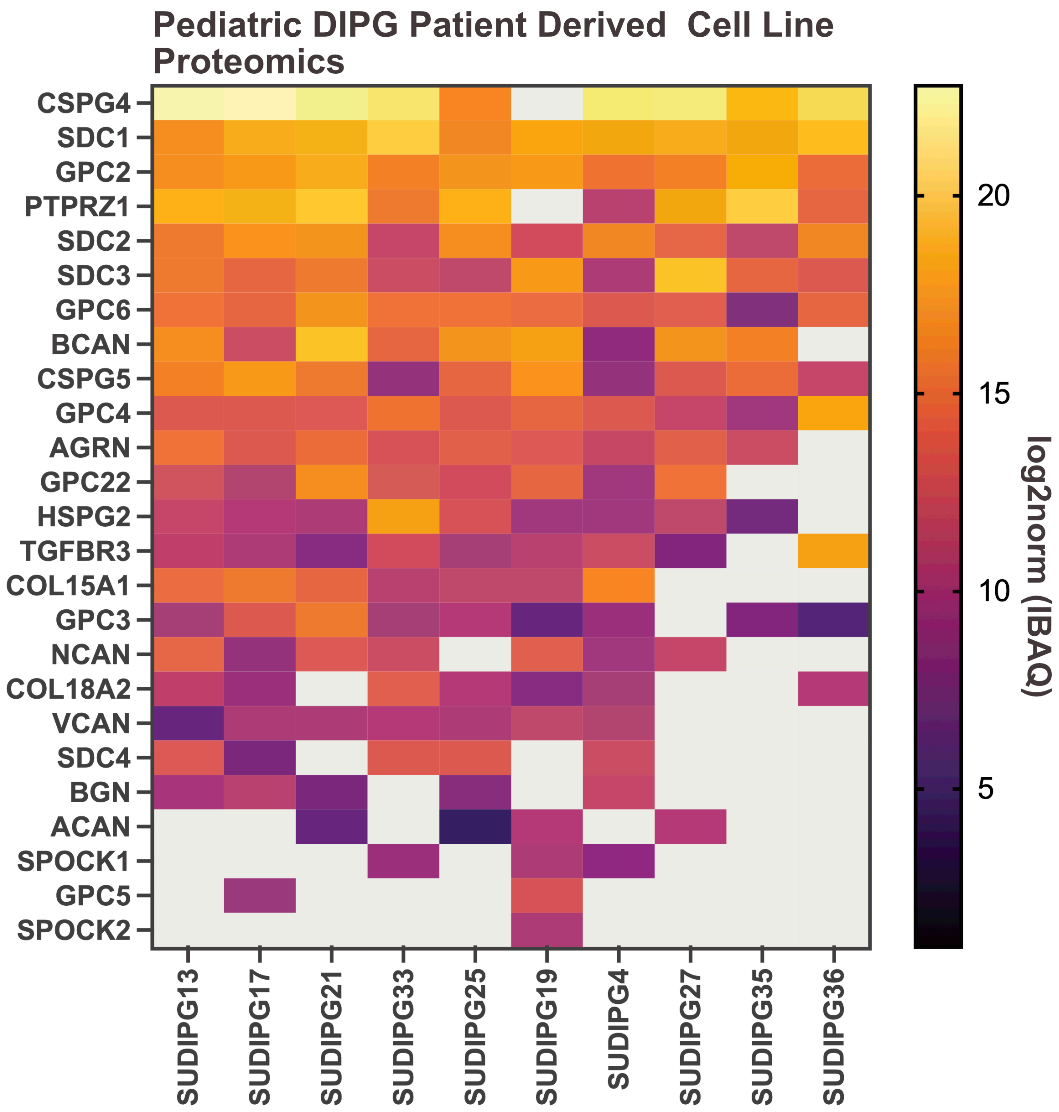

70 **Supplementary Figure 4. Presence of Heparan and Chondroitin Sulfate Proteoglycans in Patient Derived Pediatric Diffuse Intrinsic Pontine Glioma Cell Lines.** Heat map showing cell surface proteomic data, as detected by mass spectrometry, of 10 patient derived diffuse intrinsic pontine glioma (DIPG) cell lines showing the presence of heparan and chondroitin sulfate proteoglycans detected at the cell surface (grey indicates lack of detection).

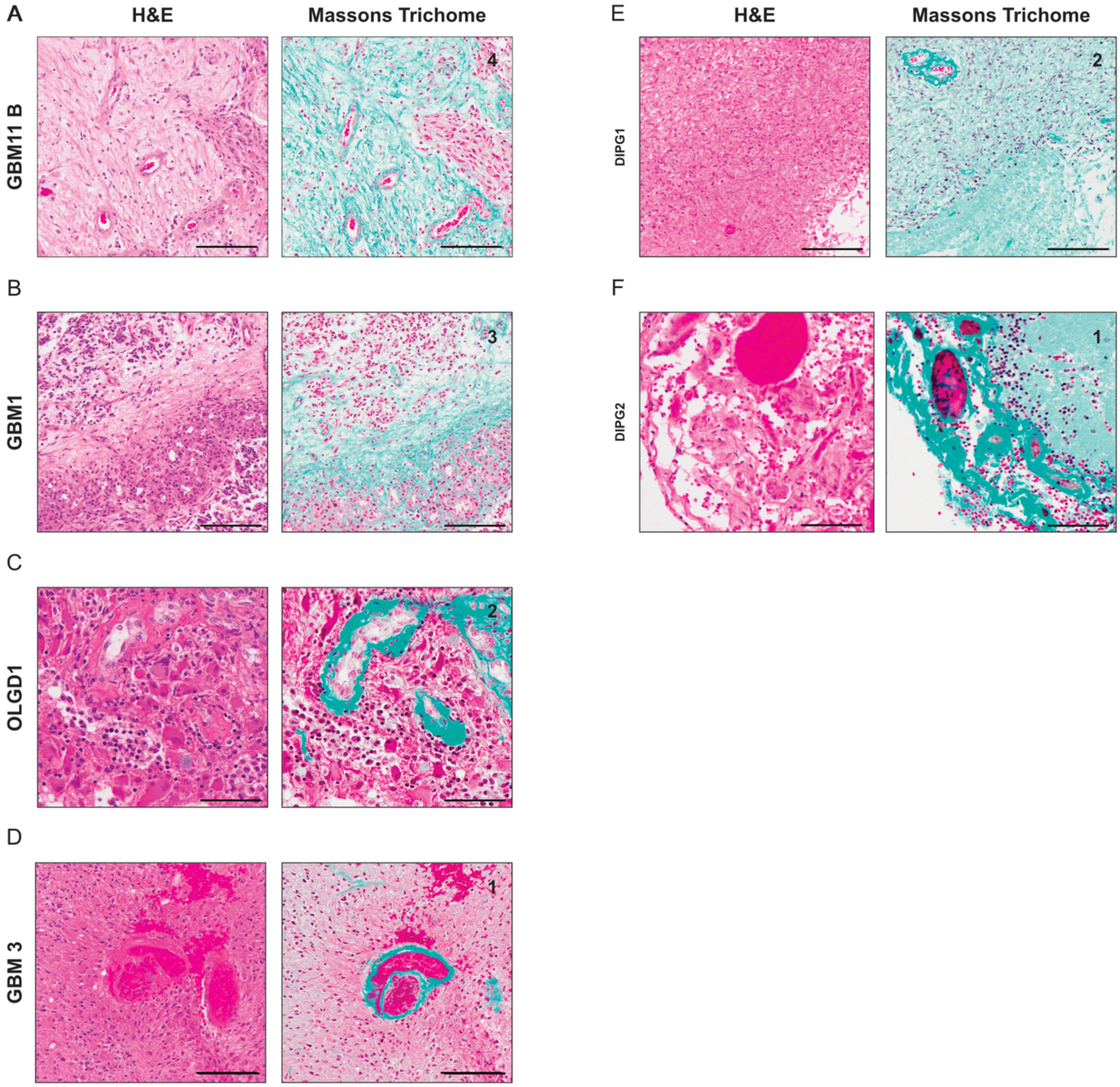

Supplementary Figure 5. Presence of Collagen in the ECM of Adult and Pediatric High Grade Glioma. Histology was performed on 45 primary Adult High Grade Glioma tumors from 30 patients and scored by an anatomical pathologist. Staining was performed on serial tumor sections, here we show Massons Trichome staining in adult high-grade gliomas ranging from tumors that scored a **(A)** 4 **(B)** 3 **(C)** 2 and **(D)** 1 (scale bar = 100 μm). Massons Trichome staining was also performed on 6 primary pediatric high grade gliomas and scored by an anatomical pathologist**.** Here we show Massons Trichome staining in pediatric high grade gliomas ranging from tumors that scored a **(E)** 2 and **(F)** 1 (scale bar = 200 μm).

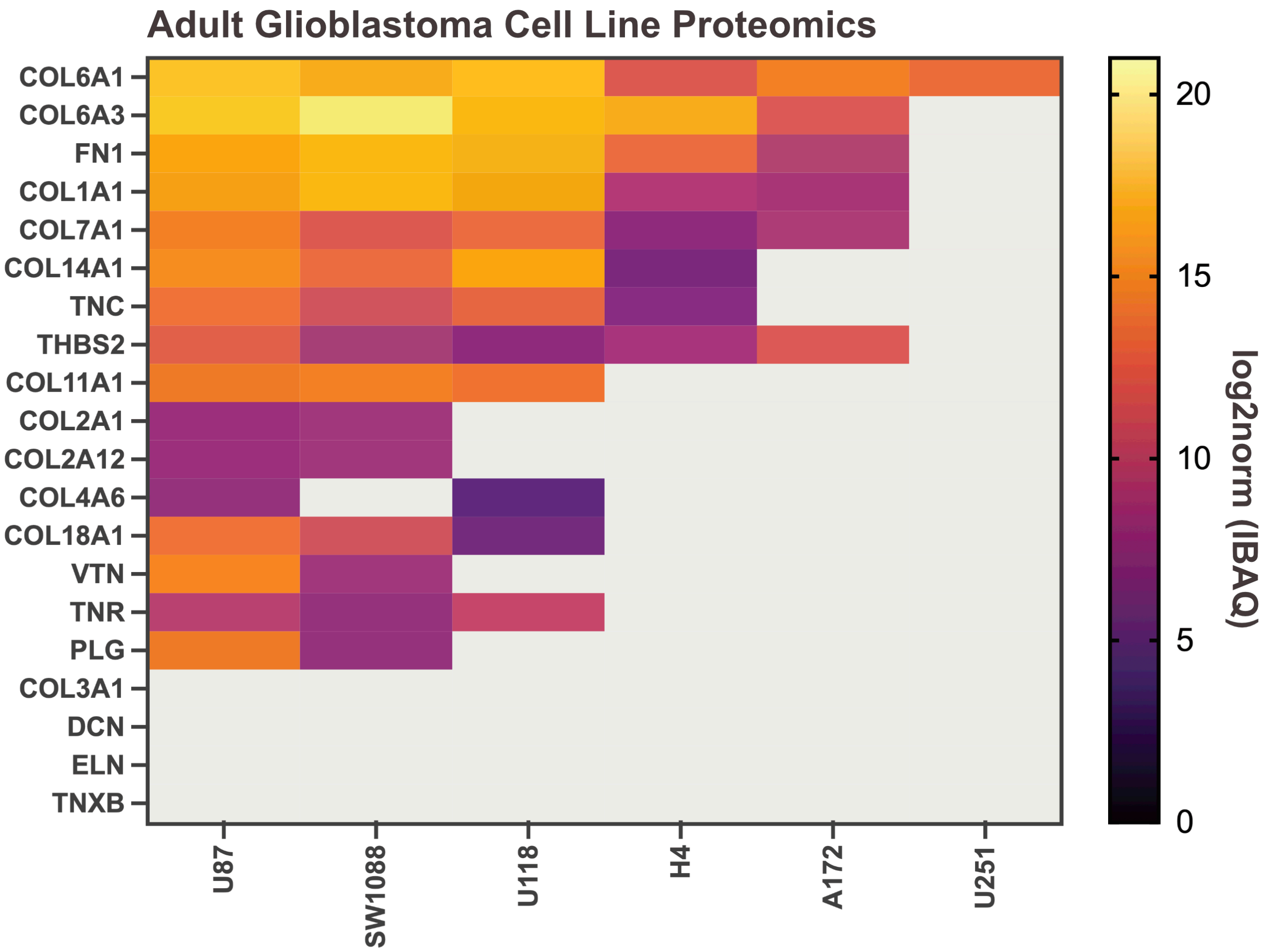

Supplementary Figure 6. Presence of Collagen and other Glycoproteins in Glioblastoma Model Cell Lines. Heat map depicting cell surface proteomic data from 6 glioblastoma cell lines showing the presence of collagen and other glycoproteins detected at the cell surface (grey indicates lack of detection).

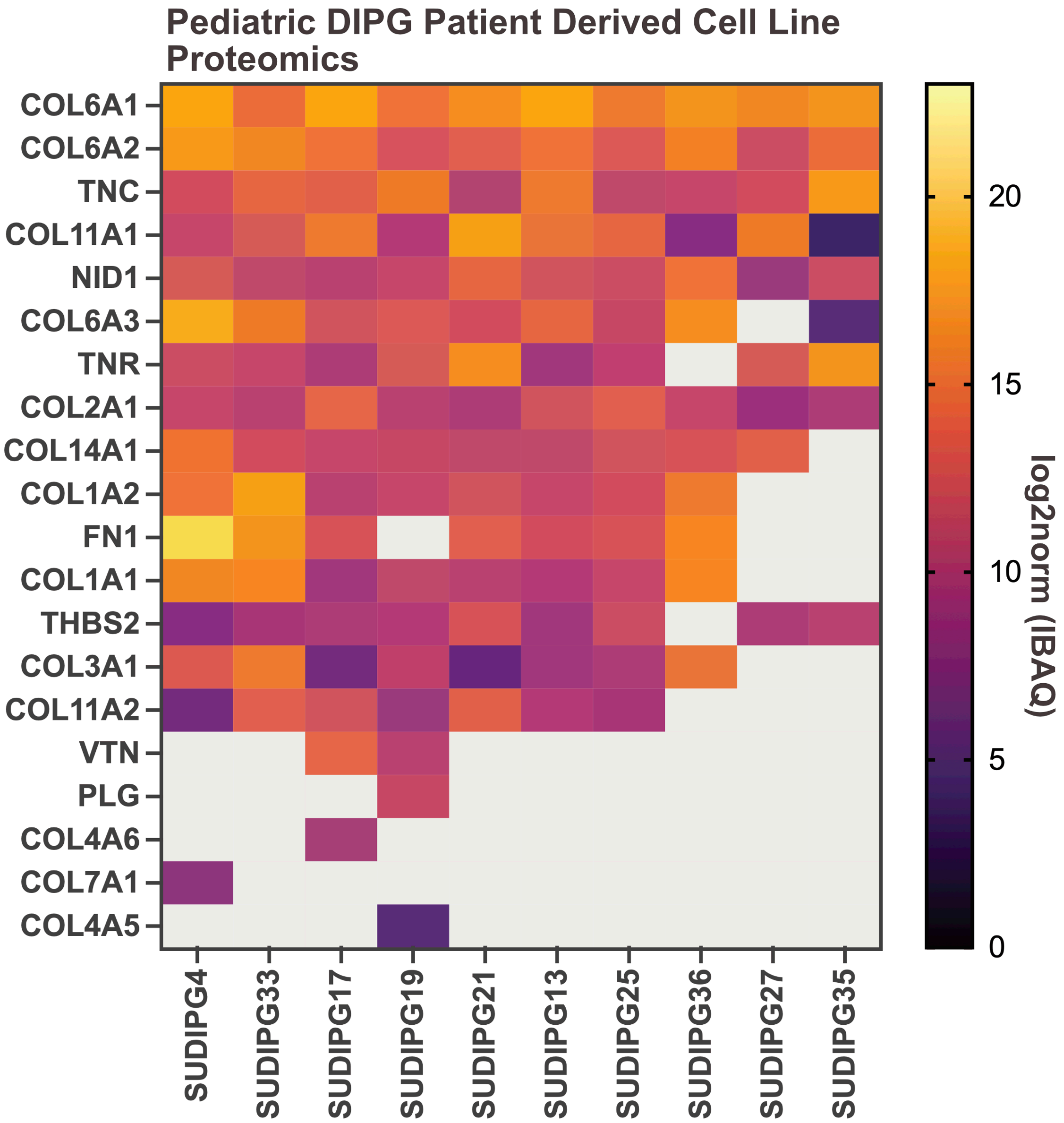

Supplementary Figure 7. Presence of Collagen and other Glycoproteins in Patient Derived Pediatric Diffuse Intrinsic Pontine Glioma Cell Lines. Heat map depicting cell surface proteomic data from 10 patient derived diffuse intrinsic pontine glioma cell lines showing the presence of collagen and other glycoproteins detected at the cell surface (grey indicates lack of detection).

Supplementary Data 1. Adult Extracellular Matrix RNAseq Data. Raw RNAseq data values from the Glioblastoma, TCGA PanCancer Atlas (n = 160 tumors).

Supplementary Data 2. Pediatric Extracellular Matrix RNAseq Data. Raw RNAseq data values from the Pediatric Brain Tumor Atlas, PBTA Provisional (n = 1945 tumors).
